# Chromosomal Redistribution of Male-Biased Genes in Mammalian Evolution with Two Bursts of Gene Gain on the X Chromosome

**DOI:** 10.1371/journal.pbio.1000494

**Published:** 2010-10-05

**Authors:** Yong E. Zhang, Maria D. Vibranovski, Patrick Landback, Gabriel A. B. Marais, Manyuan Long

**Affiliations:** 1 Department of Ecology and Evolution, the University of Chicago, Chicago, Illinois, United States of America; 2 Université de Lyon, Centre National de la Recherche Scientifique, Laboratoire de Biométrie et Biologie évolutive, Villeurbanne, France; University of Edinburgh, United Kingdom

## Abstract

Mammalian X chromosomes evolved under various mechanisms including sexual antagonism, the faster-X process, and meiotic sex chromosome inactivation (MSCI). These forces may contribute to nonrandom chromosomal distribution of sex-biased genes. In order to understand the evolution of gene content on the X chromosome and autosome under these forces, we dated human and mouse protein-coding genes and miRNA genes on the vertebrate phylogenetic tree. We found that the X chromosome recently acquired a burst of young male-biased genes, which is consistent with fixation of recessive male-beneficial alleles by sexual antagonism. For genes originating earlier, however, this pattern diminishes and finally reverses with an overrepresentation of the oldest male-biased genes on autosomes. MSCI contributes to this dynamic since it silences X-linked old genes but not X-linked young genes. This demasculinization process seems to be associated with feminization of the X chromosome with more X-linked old genes expressed in ovaries. Moreover, we detected another burst of gene originations after the split of eutherian mammals and opossum, and these genes were quickly incorporated into transcriptional networks of multiple tissues. Preexisting X-linked genes also show significantly higher protein-level evolution during this period compared to autosomal genes, suggesting positive selection accompanied the early evolution of mammalian X chromosomes. These two findings cast new light on the evolutionary history of the mammalian X chromosome in terms of gene gain, sequence, and expressional evolution.

## Abstract 2

Two bursts of gene gains occurred on the mammalian X chromosome contribute to an age-dependent chromosomal distribution of male-biased genes.

## Author Summary

Some evolutionary theories predict that the X chromosome will be enriched for genes with male functions. However, recent studies showed there had been gene traffic in which autosomal male-biased genes were retroposed from X-linked parental genes. A question remains about whether this pattern also holds for all types of new genes. Herein, using comparative genomic analysis, we dated all human and mouse genes to the vertebrate phylogenetic tree. We found that the X chromosome evolved with two bursts of gene origination events. The recent burst includes mainly male-biased genes in contrast to older X-linked genes that are often female-biased in expression. Meiotic sex chromosome inactivation contributes to this dynamic since it silences the older but not the younger X-linked genes. The older burst was after the split of eutherian mammals and the marsupial opossum, and the genes from this burst were quickly incorporated into transcriptional networks of multiple tissues, especially in the brain. The transcriptional expansion, together with the rapid protein evolution of the preexisting old X-linked genes, suggests that positive selection was acting in the early evolution of the mammalian X chromosome. These two lines of findings revealed extensive gene evolution in the mammalian X chromosome.

## Introduction

In mammals and *Drosophila*, the X chromosome usually differs dramatically from autosomes since it is hemizygous in males [1]. Sexual antagonism (beneficial for one sex, but deleterious for the other) enriches male-biased genes on the X chromosome, if alleles are generally recessive, and on the autosome if they are generally dominant [2]–[3]. On the other hand, inactivation of the X chromosome during spermatogenesis [4]–[5] drives the accumulation of male-biased genes on the autosomes where they can be expressed in the meiotic or post-meiotic phase [6]–[7]. These two processes can explain the gene traffic between the X and autosomes in *Drosophila* [8] and mammals [9]–[10] as well as the excess of male-biased genes on the autosomes [11]–[12].

However, recent analyses of male-biased genes identified several X-linked genes that originated in the last 1–3 million years (myr) in *Drosophila* [13]–[15]. Whether or not these data implicate an effect of evolutionary time on the chromosomal location of male-biased genes remains unknown. In our investigation of how the various evolutionary forces impact the chromosomal distribution of sex-biased genes, we focused particularly on how the age of genes affects their chromosomal locations. By dating when genes arose in humans and mouse, we found male-biased genes were distributed at different locations in different phases of mammalian evolution: young male-biased genes are enriched in the X chromosome, but older male-biased genes favor autosomal locations. Interestingly, this redistribution seems to be associated with feminization of the X chromosome with more X-linked old genes expressed in ovaries.

Besides the recent gene gain contributed by emergence of male-biased genes on the X chromosome, we found another burst of gene gain on X chromosome immediately after the divergence of opossum and eutherian mammals. Accelerated protein evolution and transcriptional evolution of X-linked genes reveal positive selection occurring in this period. These data support the recent notion [10],[16] that our X chromosome originated in the therian ancestor instead of the common ancestor of all mammals.

These two lines of findings significantly extend our knowledge of the origination and evolution of X chromosomes in mammals.

## Results

**Figure 1:**
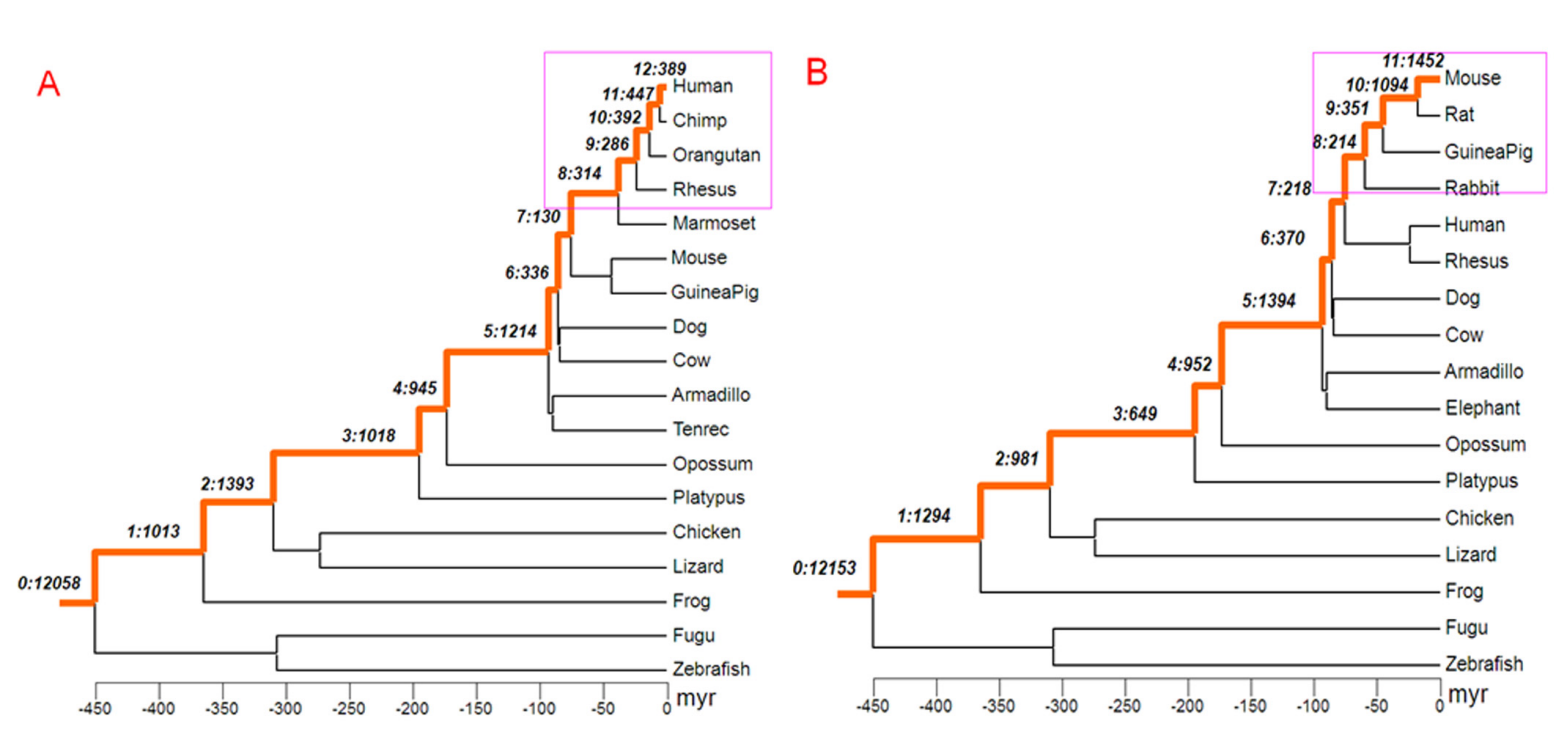
Phylogenetic tree of vertebrates with branches leading to human and to mouse marked as orange line. The topology follows UCSC genome database [17] and the species divergence time is from [18]–[24]. Panels A and B show the assignments of new genes to branches on the phylogeny of human and mouse, respectively. The young genes, those primate- and rodent-specific, are marked with pink boxes. The notation with two numbers separated by “:” indicates the branch assignment and number of genes occurring in the given branch. For example, in the human lineage represented in panel A, in branch 12, 389 genes originated after the split of humans and chimps. Due to different annotations in mouse and human, the number of genes gained may differ.

We inferred the origination times of genes based on the presence and absence of orthologs in the vertebrate phylogeny and assigned 19,935 human and 21,122 mouse protein-coding genes into different evolutionary branches (Figure 1; Table S1, S2; Materials and Methods). We found that 1,828 human genes are primate-specific (branches 8 to 12 of Figure 1A) and 3,111 mouse genes are rodent-specific (branches 8 to 11 in Figure 1B) [17]–. In subsequent analyses, except if specified elsewhere, we define them as young genes and the remaining as old genes.

Compared to previous reports [10],[25], our method identified young genes more conservatively. For example, Church et al. identified up to 2,941 primate specific genes, considerably more than we found [25]. Also, for the 67 human genes that intersect between our dataset and [10], we assigned 44 (66%) genes onto the same phylogenetic branch as they did. For the remaining 23 cases in conflict, we assigned 22 to older branches (Table S3) since we used a larger number of outgroup species.

### X Chromosome Shows Two Peaks of Gene Gain

**Figure 2:**
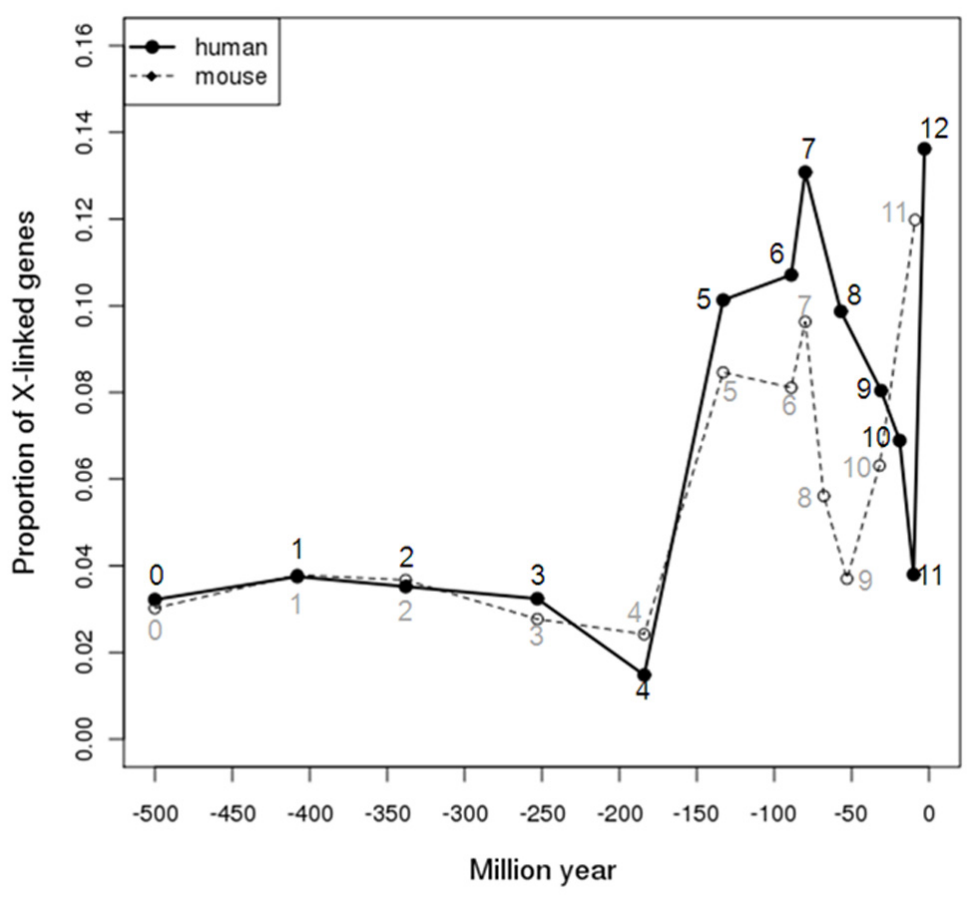
Proportions of X-linked genes arising in each phylogenetic branch. The *x*-axis indicates the age of a branch, while *y*-axis shows the proportion of X-linked genes out of all genes originating in that branch. Branch assignment in Figure 1 is labeled nearby each data point. Time in myr is calculated as the middle point of each branch. For instance, human-specific genes assigned to branch 12 are shown at −3 myr, the average origination time for an interval ranging from −6 to 0 myr. And the oldest branch (branch 0) is arbitrarily set as −500 myr.

We tracked the relative gene abundance of individual chromosomes across 450 myr and identified two bursts of genes occurring on the X chromosome (Figure 2). One burst (branches 5–7) postdated the divergence of eutherian mammals (human or mouse) and marsupials (opossum) and the other occurred recently after the split of human and chimp and after the split of mouse and rat, respectively. For both peaks, the X chromosome contributes to 8%∼14% of genes, while it only accounts for 3% of genes in the first 300 myr of vertebrate evolution. In contrast, autosomes tend to vary less in their relative contribution to the whole genome (Figure S1). As the major contributor generating new genes, DNA-level duplication accounts for 73∼95% of genes of these two peaks. If we only use DNA-level duplicates, the pattern remains the same.

Considering that many more genes arose in branch 5 compared to branch 6 or 7 (1,200∼1,400 versus 400∼500, Figure 1), the old peak seems to be best explained by the hypothesis that the X chromosome emerged in the therian ancestor and subsequently recruited many genes in an accelerated evolution of sex-related functions, as found with retrogene-based chromosomal movement studies [26]. In contrast, the recent burst reveals a rapid addition of new genes into the mammalian X chromosome, which may be independent of major chromosomal changes.

### X-Linked Young Genes Are Male-Biased, Whereas X-Linked Old Genes Are Not

**Figure 3:**
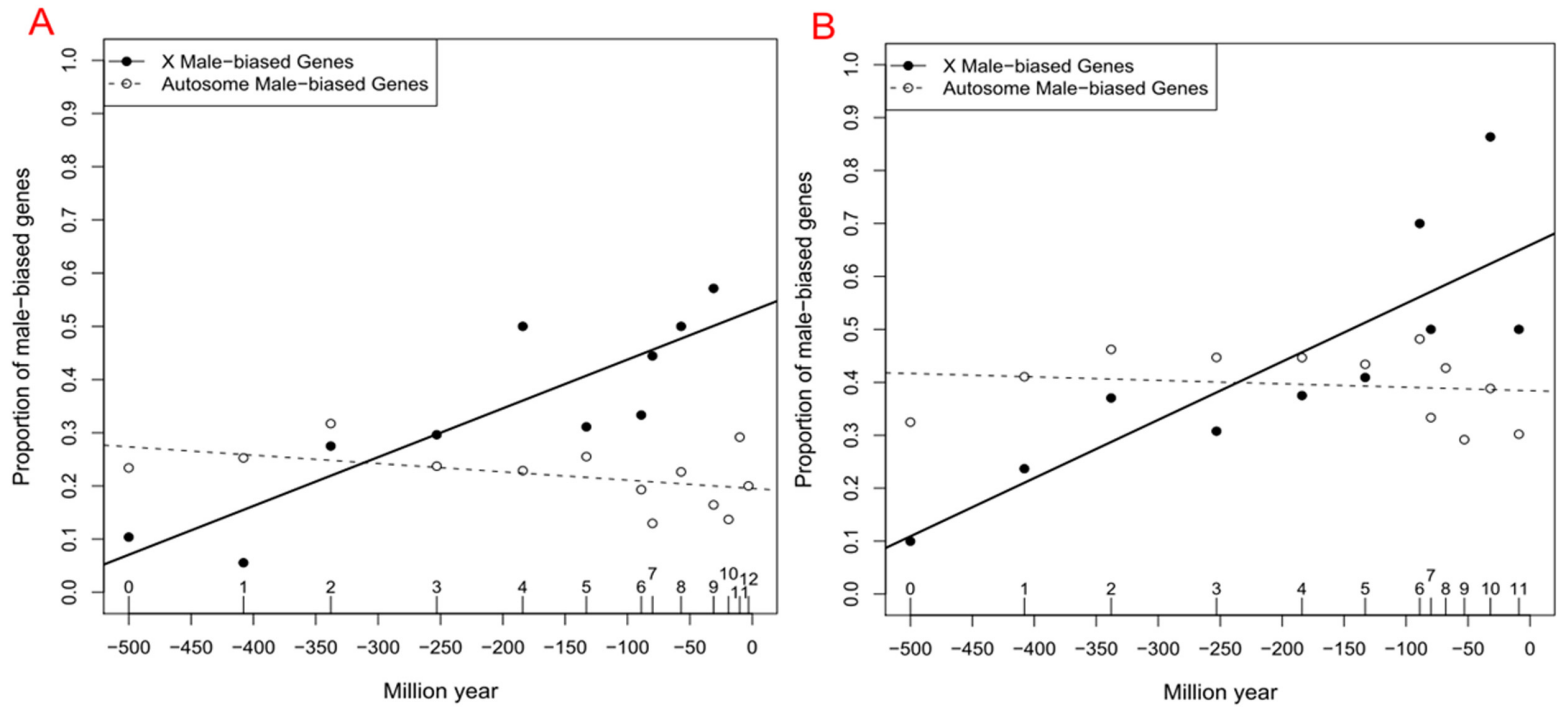
Proportions of male-biased genes out of all genes arising in each branch for the human (Panel A) and mouse lineages (Panel B). *y*-axis shows the proportions of male-biased genes out of all genes arising in that branch, whereas *x*-axis indicates evolutionary time scale (branch or million years). As in Figure 2, time in myr is calculated as the middle point for each branch. We dropped branches with fewer than five genes covered by unique Affymetrix probes such as human branch 12 for the X chromosome. The solid line and the dashed line mark the linear regressions for X chromosome and autosomes, respectively.

Based on human body index data (GSE7307, Materials and Methods) and mouse tissue profiling data [27] at the NCBI GEO database [28], we identified genes with sex-biased expression (Materials and Methods). As shown in Figure 3, both human and mouse demonstrate a similar pattern regarding the proportion of male-biased genes and the age of the branch in which they arose.

For younger branches (less than 50 myr), male-biased genes are enriched in the X chromosome compared to autosomes (∼50% versus ∼30%, Chi-square test *p*<0.05), which might be driven by fixation of recessive male-beneficial alleles under sexual antagonism. This pattern decreases for genes originating in earlier branches. Male-biased genes older than 300 myr are overrepresented on the autosomes (∼30% versus ∼15%, *p* = 1×10^−9^). This pattern was independently supported by an Affymetrix exon array panel with larger coverage of new genes (Figure S2). Thus, the recent peak observed in Figure 2 could be attributed to a burst of male-biased genes on X chromosome younger than 50 myr. Figure 3 also demonstrates that the X chromosome consists of a similar or even higher proportion of male-biased genes compared to autosomes from 90 myr ago (branch 7) to 130 myr ago (branch 5). Thus, many of the genes gained in the first, older peak may also have male-biased expression.

Notably, the proportion of female-biased genes on branch 5 was greater on the X chromosome compared to autosome (39% versus 20% in Table 1). In contrast, for branches 6 and 7, the proportion of female-biased genes is around 15% for both the X chromosome and autosomes (Table S4). Again, this suggests that the newly originated X chromosome was subjected to enhanced positive selection and recruited an excess of both male- and female-biased genes.

**Table 1:** Expression biases of genes originating in branch 5.

	Human		Mouse	
	X Chromosome	Autosome	X Chromosome	Autosome
Male	28	187	27	263
Female	35	137	26	127
Unbiased	27	409	13	216
Chi-square test *p*	2×10^−6^		0.001	

The chi-square test compares whether the X chromosome and autosomes have different distributions of expression bias.

### Positive Selection Contributes to Gene Gain on the X Chromosome

The earlier peak in Figure 2 indicates the mammalian X chromosome emerged before the divergence of eutherian and marsupial [10]. Thus, the nascent X chromosome changed remarkably, gaining an excessive number of genes. If this scenario is true, those preexisting genes on the ancestral X chromosome might have accumulated many evolutionary changes during this period (branch 5), as did genes linked to the neo-X chromosome in *Drosophila* [29]. That means we would expect these ancient genes on the X chromosome to show signatures of positive selection. To test this scenario, we investigated the evolutionary path of ancient genes shared by vertebrates by comparing the ratio between non-synonymous substitution rate and synonymous substitution rate (*Ka*/*Ks*) (Materials and Methods). In other words, we compared the *Ka/Ks* of X- and autosomal-linked old genes in separate evolutionary periods. Across evolution of 450 myr, the X chromosome did not show significantly higher *Ka*/*Ks* except in branch 5 (Table 2), which strongly corroborates the hypothesis that the X chromosome did not acquire sex-chromosome status until this period.

**Table 2:** Median *Ka/Ks* statistics for genes occurring before vertebrate split (genes assigned to branch 0 in Figure 1) along their evolutionary path from the common ancestor to human lineage.

Branch^a^	X Chromosome^b^	Autosome	Wilcox Single-Tail Test *p* c
3	0.073 ([0.019, 0.186])	0.061 ([0.0202, 0.165])	0.357
4	0.059 ([0.014, 0.414])	0.067 ([0.015, 0.294])	0.559
5	0.057 ([0.029, 0.110])	0.047 ([0.021, 0.093])	0.035
6	0.015 ([1e^−4^, 0.131])	0.014 ([1e^−4^, 0.146])	0.595
7	1e^−4^ ([1e^−4^, 0.087])	1e^−4^ ([1e^−4^, 0.129])	0.841
8	0.026 ([1e^−4^, 0.073])	0.036 ([1e^−4^, 0.089])	0.916
9	1e^−4^ ([1e^−4^, 0.062])	1e^−4^ ([1e^−4^, 0.102])	0.946
10	1e^−4^ ([1e^−4^, 0.073])	1e^−4^ ([1e^−4^, 0.097])	0.889
11	1e^−4^ ([1e^−4^, 0.109])	1e^−4^ ([1e^−4^, 0.089])	0.219
12	1e^−4^ ([1e^−4^, 0.115])	1e^−4^ ([1e^−4^, 0.184])	0.966

Only alignments with at least 300 bps were used.

a Branch 1 is excluded from this analysis since we could not get a polarized *Ka/Ks* given a single outgroup (fish); Branch 2 was also excluded since we often lacked sequences for the frog possibly due to its fragmentary assembly.

b The median value together with the 25% and 75% quantile value are shown.

c We used one-sided Wilcoxon rank sum test to check whether X-linked genes show higher *Ka/Ks* compared to autosomal ones. Thus, if *p* is close to 1, then the autosome has bigger *Ka/Ks*, as seen in branch 8.

We extended this analysis to genes gained since branch 5. We directly estimated the proportion of replacement substitutions (*α*) based on polymorphism and divergence data in [30] and a maximum-likelihood method implemented in the DoEF package [31]. As shown in Table S5, young genes generally show higher *α* compared to old genes, and X-linked male-biased genes show the highest *α*, 0.501. This pattern shows that positive selection instead of neutrality drives the evolution of X-linked genes arising since branch 5, especially those with male-biased expression.

However, positive selection of nucleotide substitutions can only suggest that initial fixation may also be driven by positive selection. More direct evidence comes from copy number polymorphism (CNP) data in *Drosophila*, which showed that the X chromosome is subject to stronger purifying selection than autosomes [32]. In human, it was also noted that the X chromosome shows a paucity of CNPs [33]. Together with bursts of adaptive fixations occurred on the neo-X of *Drosophila* [29], it is likely that positive selection instead of drift accounts for two bursts of genes on the X chromosome.

### Male-Specific Chromosomal Inactivation May Account for Age-Dependent Distribution of X-Linked Male-Biased Genes

As we noted before, enrichment of young male-biased genes on the X declines for those originating in earlier evolutionary branches. Using expression data from mouse spermatogenesis, we compared different age groups to investigate which force underlies such a demasculinization process (Table 3). As previous studies such as [7] found, old genes are expressed more in the pre-meiosis stage (spermatogonia) but are silent from meiosis (pachytene spermatocyte) to post-meiosis (round spermatid). In terms of whole testes, however, old X-linked genes are underrepresented (Table 3). New genes show a distinct pattern: while often expressed in spermatogonia, they are not silent in meiosis. Moreover, a much greater proportion of new genes on the X are expressed in the post-meiosis stage compared to genes on the autosome (70% versus 27%, Chi-square test *p* = 5×10^−10^). This is consistent with a previous observation of X-linked postmeiotic multicopy genes [34], the vast majority of which we found were very young (Materials and Methods). Such a pattern suggests that the young X-linked genes are not affected by MSCI. An independent microarray dataset of mouse spermatogenesis [35] confirms high expression of X-linked young genes in spermatid (Figure S3). In addition, we note that the customized array by Khil et al. was comprised mainly of old and conserved genes, with only 1.7% of the set being young genes. In contrast, the Affymetrix array data [36] we used covered 14,923 Ensembl genes, 3.9% of which are young genes.

**Table 3:** Distribution of expressional presence across spermatogenesis.

	Type A Spermatogonia	Type B Spermatogonia	Pachytene Spermatocyte	Round Spermatid	Whole Testis^a^
**Old genes**					
X (489)^b^	319 (69%)^c^	318 (70%)	157 (36%)	218 (49%)	234 (57%)
Autosome (13,847)	8,157 (64%)	8,403 (67%)	7,454 (57%)	7,363 (57%)	8,008 (65%)
Chi-square test^d^	*p* = 0.01	*p* = 0.16	*p*<2.2×10^−16^	*p* = 0.002	*p* = 0.002
**Young genes**					
X (35)	11 (33%)	11 (37%)	9 (29%)	23 (70%)	22 (71%)
Autosome (552)	92 (18%)	105 (22%)	118 (23%)	139 (27%)	139 (29%)
Chi-square test	*p* = 0.06	*p* = 0.09	*p* = 0.56	*p* = 5×10^−10^	*p* = 3×10^−6^

a For comparison, we called expressional presence in four different cells based on the data generated in [36] and in the whole testis based on [27], respectively.

b The X chromosome encodes 489 old genes and 35 young genes covered by unique probes on the Affymetrix Mouse Genome 430 2.0 Array.

c Out of 489 X-linked old genes, 319 are called present in both replicates, 141 are called absent in both replicates, and 29 are ambiguous. We called the proportion as 319/(319+141) or 69%.

d We performed a chi-square test of whether the proportions of genes expressed in one cell type differs between X chromosome and autosomes.

**Figure 4:**
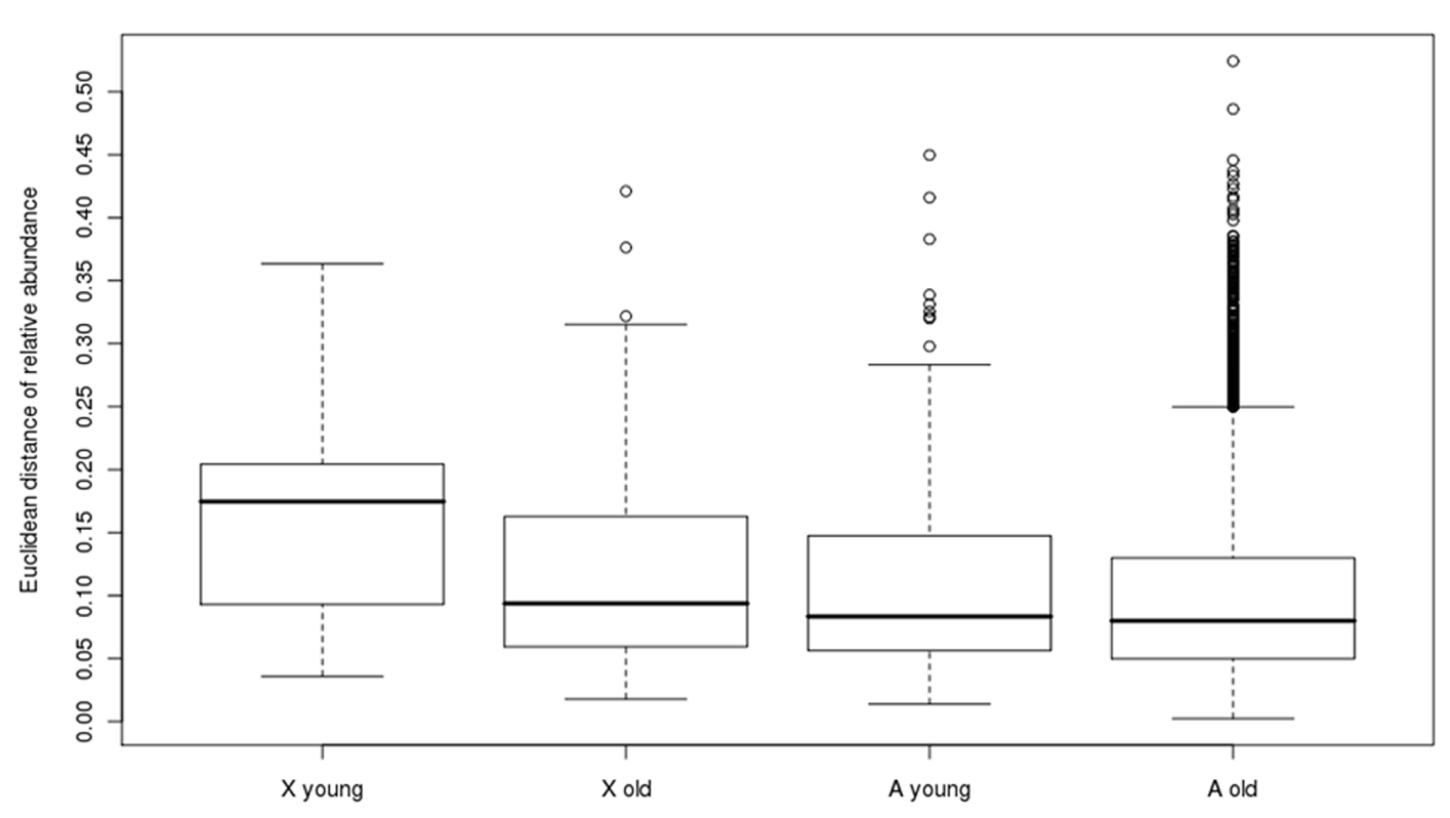
Expression divergence between mouse and rat testes. Since rodent specific genes with unique probes in both mouse and rat are too few, here we define genes emerging since branch 5 as young genes and the remaining entries as old genes.

This striking contrast between young and old genes suggests that MSCI plays an important role in determining the age-dependent chromosomal distribution of male-biased genes. In order to investigate how this contrast occurred in such a short time, we analyzed four major cell types including sertoli cells, spermatogonium, spermatocyte, and spermatid between mouse [35] and rat [37]. We used the Euclidean distance of relative abundance (RA) to measure how orthologous genes have diverged in their expression (Materials and Methods). Consistent with a previous comparison of human and chimpanzee [38], the testis expression of genes on the X chromosome diverge more between rat and mouse than genes on autosomes (Wilcoxon rank sum test *p* = 4×10^−6^, Figure 4). Furthermore, X-linked young genes show significantly higher divergences, compared to all other three groups (*p*<0.05).

While we found that expression in various spermatogenesis stages is generally conserved [35] with only about 3% divergence (Figure S4), X-linked young genes show the largest expression divergence in spermatid. Specifically, after the split of mouse and rat 37 myr ago [39], young X-linked genes show 6.9% divergence in spermatid, which is much higher than the genomic average for spermatid, 3.3% (Wilcoxon rank sum test *p* = 0.002). This increased divergence suggests that, although these genes seem to escape MSCI and preferentially transcribe in post-meiosis, the expression profile is not conserved. It remains unknown whether these genes get up-regulated or down-regulated in one species. But if the latter case were true, it indicates that the high post-meiotic expression would be silenced by MSCI in later evolution. This could also explain how the different pattern between young and old genes in Table 3 is achieved.

### Feminization of the X Chromosome Over Evolutionary Time

We investigated the distribution of female-biased genes on chromosomes and its correlation with gene ages. Interestingly, female-biased genes are distributed in a pattern symmetrical to male-biased genes (Figure S5 versus Figure 3): the old X-linked genes are more often female-biased, while young genes are not.

**Figure 5:**
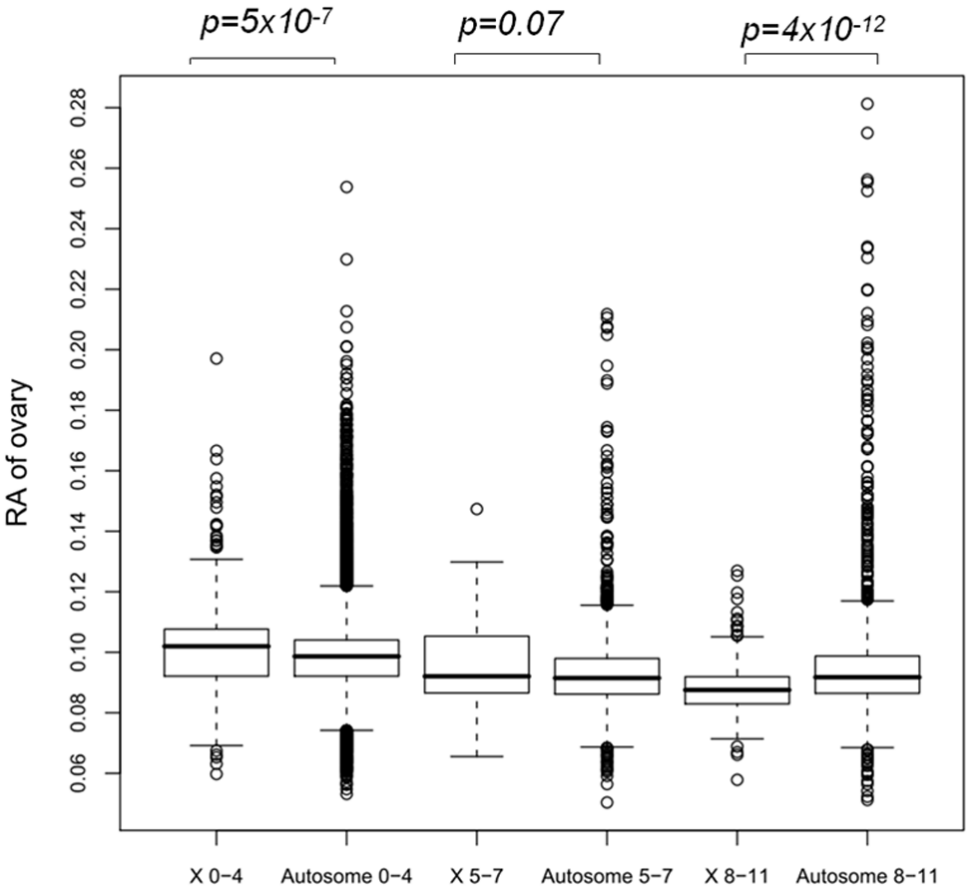
Distribution of relative abundance (RA) in ovaries across genes from distinct age groups. We binned 12 branches into three major groups: before the emergence of the mammalian X chromosome (branch 0–4), rodent lineage (branch 8–11), and the middle interval (branch 5–7). Wilcoxon rank sum test is used to investigate whether ovary expression is identical between autosome and the X chromosome.

We characterized ovary expression of genes using the Affymetrix mouse exon array panel data. Consistent with Figure S5, ovary expression also depends on the age of the gene's origination. Specifically, young autosomal genes show significantly higher expression in ovaries than young X-linked genes (Wilcoxon rank sum test *p* = 5×10^−12^, Figure 5). However, old X-linked genes generally show higher expression in ovaries (*p* = 5×10^−7^). Thus, as gene age increases, this expressional excess of autosomal genes reverses and older X-linked genes show significantly higher expression in ovaries.

It can be argued that such an age-dependent pattern of expression is not a specific property of ovary evolution and other organs might also show a similar pattern. To test this possibility, we investigated gene expression in the major organs: brain, heart, kidney, liver, lung, muscle, spleen, and thymus. All these tissues, except for brain, showed a significant excess of expression for new genes (branch≥5) on autosomes compared to that of X-linked genes (Wilcoxon rank sum test *p*<0.01, Figure S5). However, for old genes (branch≤4), they are evenly distributed (*p*>0.05).

The brain shows a unique pattern. Young genes (branch>7) are relatively abundant on autosomes (*p* = 0.001, Figure S5), but old genes (branch≤7) are overrepresented on the X chromosome (*p*≤0.01). This is consistent with previous findings that X chromosome is enriched with genes expressed in brain [1],[40]. Notably, different from ovaries, enrichment in the brain did not show clear age dependence, since genes originating from branches 5 to 7 presented the most significant excess (Figure S6).

### A Transcription Burst Occurred for X-Linked Genes Originating in Branch 5, Which Is Most Pronounced for Brain

The coincidence that the X chromosome is enriched with both ovary-expressed and brain-expressed genes occurring in branch 5 (Table 1; Figure S5) motivated us to perform more thorough transcriptional profiling to get a more complete picture of how genes from this evolutionary period are transcribed.

**Figure 6:**
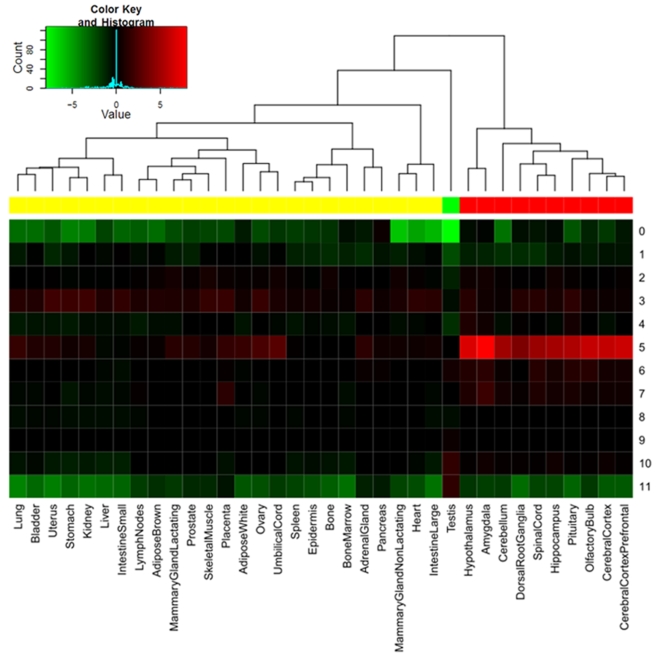
Heatmap of expression enrichment in the X chromosome versus autosomes based on mouse tissue profiling data. The column indicates different tissue samples, while the row indicates different evolutionary branches. For each cell, we performed a Fisher-exact test on whether the X chromosome and autosomes have similar proportions of genes expressed in this tissue. Then, we converted the *p* into unsigned log10 based values. We added a negative sign for cells if autosomal genes are more enriched. So a value of 2 means that expression for X-linked genes is significantly overrepresented for this cell. Notably, we arbitrarily set one exceptional excess of autosomal testis genes on branch 0 (*p*∼10^−22^) as 10^−8^ to make the excess on autosome and X chromosome symmetric. The dendrogram and top side color bars mark the three different groups of tissues.

We investigated mouse exon atlas data (GSE15998) to ask whether X-linked genes are more frequently expressed in the tissue of interest across different age groups. We clustered tissues by the proportion of X-linked genes expressed versus the proportion of autosomal genes expressed and identified three major groups: nervous system, testes, and all other tissues (Figure 6). Remarkably, the X-linked genes originating in branch 5 are transcriptionally permissive with a larger proportion of them expressed in many tissues compared to autosomal genes. This excess is most pronounced for brain samples.

Consistently, human data revealed that a greater proportion of X-linked genes emerging on branch 5 are expressed more widely than autosomal genes originating in this period, which is strongest for the brain (Figure S7). Since human and mouse share a similar pattern, parsimony suggests this striking transcriptional pattern of branch 5 derived genes is ancestral. Notably, none of these genes show sex bias in human brain profiling data [41], which suggests they might be important for both sexes.

### Origination Patterns of miRNAs Are Similar to Protein Coding Genes

We have described evolutionary patterns of protein-coding genes, which could be driven by natural selection in various forms like sexual antagonism or MSCI. If, however, such a pattern is a product of some mutational bias of gene origination, we would not detect similar evolutionary patterns in non-coding RNA genes, such as X-linked miRNAs. Therefore, we investigated the chromosomal distribution of miRNA genes annotated in miRBase [42] and found that miRNA duplicates are distributed in a pattern similar to that observed for protein-coding genes (Table S6). Specifically, both human and mouse show significant miRNA gene gain in branches 5 to 7 compared to the proportion of all miRNA genes (18∼22% versus 10∼13%, Fisher's Exact Test *p*<0.05). Moreover, they also show an excess for the youngest branch. Although it is not significant for the human data due to small sample size, it is for mouse (*p* = 0.02).

Like protein-coding genes, a larger proportion of X-linked miRNAs originating in branch 5 are transcribed in nine tissues (statistically significant for six of them) surveyed on Agilent chip [43] compared to autosomal genes (Table S7; Materials and Methods). Moreover, semi-quantitative PCR data of X-linked miRNAs in 12 tissues [44] show 9 out of 13 (69%) young genes are expressed higher in testes than at least six non-testis tissues. However, this percentage drops to 23% for old X-linked genes (9 out of 39, Fisher's Exact Test *p* = 0.005). Consistent with protein-coding genes, these data also show that old genes have moderate or high expression in ovaries and the young genes show only trace levels of expression (Wilcoxon rank sum test *p* = 0.01).

The age-dependent locations and expression profiles of miRNAs support that it is evolutionary forces, rather than some mutation bias intrinsic to a certain type of gene, which account for the dynamics of X-linked gene evolution.

### The Temporal Pattern of Gene Origination Is Consistent with the Evolutionary History of the X Chromosome

**Figure 7:**
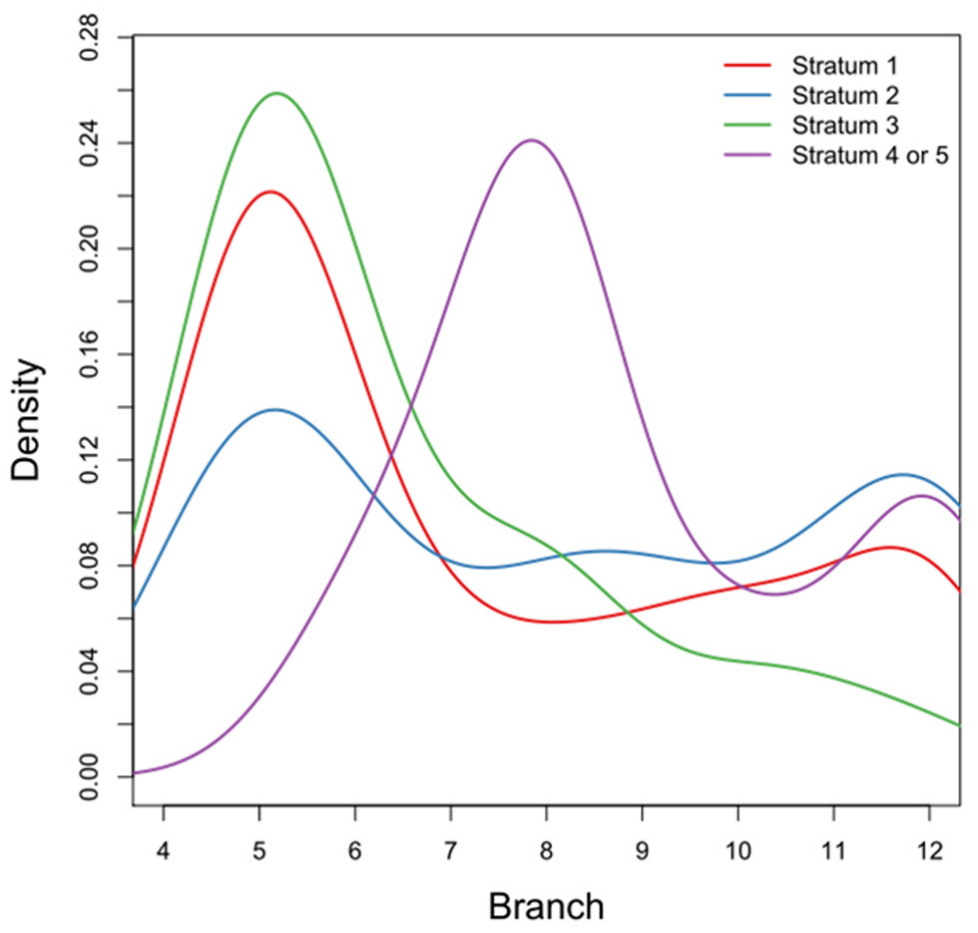
Density plot of genes with respect to branches and strata. The stratum border information is from [49].

It is known that the X chromosome can be divided into five evolutionary strata because of step-wise repression of recombination [45]–[47]. The X-conserved region (XCR) consists of the oldest strata 1 and 2, while the X-added region (XAR) includes younger strata 3 that is shared by primates and rodents, and much younger 4 and 5 that were derived within primates [46],[48]. Since sexual antagonism or other sex related forces like the faster-X process (see Discussion) depends on hemizygosity of the X chromosome in male, we expect the accordance between bursts of gene gain with the formation of corresponding strata. If these forces shape the evolution of gene content on the X chromosome, we should find that X-linked genes originating at a given time period should accumulate only in the strata already formed at that time. In other words, we should find a correlation between the ages of genes and the strata in which they are located. Consistent with these predictions, Figure 7 shows that the older strata 1 to 3 are associated with relatively older genes, while strata 4 or 5 are enriched with younger genes (one sided Fisher's Exact Test *p* = 0.03). This finding parallels the temporal correspondence between the occurrence of strata and the out-of-X retrogene traffic [49].

## Discussion

### Remodeling of the Newborn X Chromosome

Our analyses demonstrated that the X chromosome evolved dramatically on both the sequence and expression levels after the split of eutherian mammal and marsupials. Specifically, the X chromosome showed a burst of gene gain during this time, and many of these genes quickly invaded the transcriptional network of various tissues, especially the brain. Furthermore, genes predating the birth of the X showed rapid protein-level evolution. A straightforward interpretation is that the newborn mammalian X was subjected to strong positive selection similar to the neo-X chromosome in *Drosophila* [29]. Moreover, the X-linked genes arising in branch 5 seem to have played important roles, as shown by their broad expression. Their transcription pattern suggests that the early evolution of placental mammals was associated with rapid changes in the brain. Furthermore, analysis of gene ontology showed that many of these genes mainly played regulatory roles in transcription and metabolism (Table S8). Thus, regulatory change contributed by gene gain on the X chromosome was extensively involved in the initial evolution of eutherian mammals. The fact that this peak ranges between branches 5 and 7 suggests remodeling of incipient X chromosome might take about 90 myr (−160∼−70 myr, Figure 2), which is consistent with one report based on retrogene movement [26]. However, the selective pressures driving this dramatic change in branch 5 appear to be smaller in subsequent branches (Table 2).

### Redistribution of X-Linked Male-Biased Genes

Our analyses reveal chromosomal redistribution of X-linked male-biased genes. Sexual antagonism may contribute to the initial fixation of X-linked recessive alleles as described previously [2],[7]. The faster-X hypothesis was initially proposed to fix more mutations on the X chromosome only if they are recessive and beneficial [1]. Recently, it was observed that this force was most pronounced for male-biased genes [50]. This suggests that the faster-X process could also be involved in the emergence of young X-linked male-biased genes, as the hypothesized sexual antagonism might.

These young X-linked male-biased genes could be later silenced by MSCI as suggested by Table 3, Figure 4, and Figure S4. At least two processes could be involved in this switch. First, we found a statistically significant excess of male-biased retrogenes generated in the X→A movement process and X-enrichment of the female-biased parental genes for both human and mouse (Table S9). Thus, the demasculinization and feminization of the X chromosome could be coupled in retrogene traffic. Moreover, our RA analysis (Figure S6) extends the out-of-the-testes hypothesis [51] to non-retroposed new genes. We found that new genes generally acquire transcription in more tissues during evolution although they are initially enriched in testes. With increasing MSCI and expanding expression breadth, X-linked male-biased genes might become unbiased or even female-biased as Figure S6 shows.

### Spatial Distribution of X-Linked Male-Biased Genes

If new strata on the X chromosome represent regions that did not develop recombination repression until recently, the genes encoded in these regions will often escape MSCI [45]. Thus, it is expected that the X-linked male-biased genes more likely escape MSCI when located on young strata or pseudoautosomal regions (PARs). However, out of 13 young male-biased genes in humans, the relatively young strata 4 and 5 encode only one (Table S10), which does not significantly differ much from the expected number based on its genomic size. How then did the remaining 12 genes, those situated on older strata, escape from MSCI?

It was proposed that the excess of inverted repeats (IRs) encoded by human and mouse X chromosome could protect genes contained by these IRs from MSCI [52]. IRs suppress MSCI through formation of cruciforms or other unusual chromatin structures. Moreover, cancer/testis (CT) genes that are often expressed in normal testes and in cancerous tissues frequently overlap with IRs [52]. Given that X-linked CT genes underwent recent expansion [53], it is not surprising that some of them could form highly homologous IRs. In fact, 8 out of 13 young X-linked male-biased genes are CTs (Table S10). Thus, the high IR abundance on the mammalian X chromosome might be one reason that these genes can be transcribed in meiosis or postmeiosis.

Furthermore, out of 12 genes encoded by PARs and covered by unique probes (Table S11), there is only one (8%) male-biased gene, *PPP2R3B*, which is shared by human and mouse. Thus, different from our intuition, PARs do not harbor an excess of male-biased genes compared to the remaining strata (18%) and to autosomes (24%). Albeit of small sample size, this observation suggests that sex-related forces like sexual antagonism or faster-X process account for the observed excess of young X-linked male-biased genes.

There are only limited number of genes with unique probes on strata 4 (five) and 5 (eight). For the remaining strata, stratum 3 is enriched with male-biased genes, which is much higher than stratum 1 (27% versus 17%, one-sided Fisher's Exact Test *p* = 0.02) and stratum 2 (27% versus 15%, *p* = 0.03). This pattern suggests that stratum 3 recruits more young male-biased genes and there was not enough evolutionary time to be feminized as occurred in the oldest strata 1 and 2.

### Rodents Likely Undergo Stronger Selection

As shown in Figure 2, the emergence of young male-biased genes peaks in recent evolution of human and mouse. However, this peak started 30 myr ago (before the divergence of mouse and rat) in the rodent lineage, while the peak appeared in the last 5 myr in human lineage.

This difference is consistent with the fact that the mouse X encodes more young male-biased genes than the human X. Specifically, male-biased genes account for 52% and 74% of X-linked young genes in human and mouse, respectively (Figure 3; one sided Fisher's Exact Test, *p* = 0.07). Exon array data are similar (Figure S2; 45% versus 76%, one sided Fisher's Exact Test, *p* = 2×10^−8^). Origination of significantly more male-biased young genes suggests that stronger positive selection acts on rodents and could explain why the recent peak of gene gain (Figure 2) began earlier in the mouse lineage than in the human.

## Materials and Methods

We downloaded Ensembl [54] release 51 (November, 2008) as the basic gene dataset for our analyses. We used MySQL V5.0.45 to organize the data, BioPerl [55] and BioEnsembl [56] to fold the pipeline, and R V2.8.0 [57] to perform all statistical analyses.

### Dating Human and Mouse Genes on the Vertebrate Phylogenetic Tree

We developed a genome-alignment based pipeline to infer the origination time of a given genomic region by modifying a previous gene-alignment based method [58]. We analyzed UCSC [17] netted chained file for human (hg18) and mouse (mm9) to verify whether a given human/mouse locus has a reciprocal syntenic alignment in the outgroup genome such as chimpanzee, rat, chicken, and so on. In other words, we investigated whether a best-to-best match could be found between human/mouse loci and outgroup loci regardless of chromosomal linkage. In this way, we can identify orthologous genes; even those with different chromosomal location due to fusions or translocations such as those found in XAR region will be identified as well. Then, in order to handle occasional sequencing gaps, we scanned multiple outgroups and assigned this locus to a specific branch by following a parsimony rule. Compared to the previous method [58], our strategy is independent of gene annotation of outgroups and robust with gene translocation. Thus, we generated a more stringent young gene dataset (as described in the Result section). And, as Figure S8 shows, we have not assigned most genes encoded by XAR as young genes simply because this region changed the linkage by fusing to X chromosome. Conversely, several genes originated in branch 5 are located in strata 1 and 2 that are not XAR (Figure S8), also supporting that our pipeline is robust with gene translocations.

Notably, for regions without reliable synteny, our method might not work. This situation would be most pronounced for telomeres, which tend to be repetitive and prone to recombine [59] and thus have very limited synteny. For example, we dated 17 genes situated on PARs of the X chromosome (Table S11). For three genes encoded by PAR2, repeats contribute less than 16% of the gene loci based on UCSC annotation [17]. Accordingly, our age assignments for these three genes are always consistent with those inferred by tree reconstruction provided by Ensembl [54]. In contrast, for 14 genes linked with PAR1, repeats are prevalent with a median contribution of 55% to the gene loci. In this case, our results are consistent for only three out of nine cases with Ensembl age information.

We slightly modified the previous pipeline [58],[60]–[61] and classified young genes as DNA-level duplicates, RNA-level duplicates (retrogenes), and de novo genes. Briefly, we performed all-against-all BLASTP search for human and mouse proteins. It was reported previously that retrogenes can recruit other neighboring genome regions with introns after being retroposed [51]. Thus, in order to define a new gene as retrogene, we requested that in the aligned region between the most similar paralog (candidate parental gene) and child genes, the former contain at least one intron and the latter to be intronless. Otherwise, it will be classified as DNA-level duplicates. Notably, if there is no hit with BLAST evalue cutoff 10^−6^ found [58] and no annotated paralog by Ensembl [54], the gene will be defined as de novo.

### Expression Profiling

In order to avoid non-specific probes and to cover more recently annotated genes, we used the customized array annotation files (released on November, 2008) downloaded from University of Michigan [62], HGU133Plus2_Hs_ENSG (Affymetrix Human 133 plus 2) and Mouse4302_Mm_ENSG (Affymetrix Mouse Genome 430 2.0 Array) for human and mouse, respectively. For exon array analysis, we used HuEx-1_0-st-v2,U-Ensembl49,G-Affy.cdf and MoEx-1_0-st-v1,U-Ensembl50,G-Affy,EP.cdf generated by Aroma.affymetrix team [63]. Thus, we excluded some candidate young genes that were too similar to their paralogs and did not have specific probes.

Based on R [57] and Bioconductor platform [64], we used RMA [65] to normalize and generate gene-level intensity for 3′ gene array and Aroma.affymetrix to normalize and summarize gene-level signal for exon arrays. We used MAS5 to call expressional presence and absence for 3′ gene array. In case of exon array, we used Affymetrix dabg (detection above background) algorithm to generate chip specific background signal and then compared gene-level signal to this background with Wilcoxon rank sum one-tail test. Considering multiple-testing issues, we converted all *p* values to *q* values using the qvalue package [66]. The *q* value of 0.01 was used as the cutoff. For Agilent miRNA array, we used “gIsGeneDetected” column generated by Agilent Feature Extraction software to define presence or absence calls [67]. We required a gene to be present in all replicates to be considered a presence and a gene to be absent in all replicates to be considered an absence. We removed all ambiguous cases from the final statistics.

We used the LIMMA package [68] to call expressional difference, with a false discovery rate corrected *p* of 0.05 used as the cutoff. Although we compared testis and ovary, we used the term “male-bias” or “female-bias” rather than “testis-bias” or “ovary-bias.” The reason is that these two datasets are nearly equivalent. A previous study showed that the proportion of germline male-biased genes is much higher than that of somatic male-biased genes (20% versus 2%) [12].

For meta-analyses of mouse and rat spermatogenic data, we followed the concept of RA and euclidean distance (*d*) to measure the between-species expression divergence [69]. Specifically, we defined RA as the proportion of expression intensity of one tissue out of all tissues and *d* as the sum of the square of RA difference for all tissues between mouse and rat, i.e., 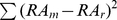.

We mapped 20 out of 33 representative genes in [34] to our gene age data using unique NCBI gene names. Remarkably, 16 (80%) are rodent-specific, with 11 of them originating after the mouse and rat split. We note here that this dataset does not overlap with what we described in Table 3, since Table 3 only presents genes with unique probes, which 19 of these 20 genes do not have.

### Branch-Specific *Ka/Ks* Analysis

We downloaded the vertebrate-wide 44-way coding sequence alignment from UCSC. UCSC known genes mapping to multiple Ensembl genes were discarded. For Ensembl genes mapping to multiple UCSC known genes, we retained only one UCSC gene with the longest coding region. Then, considering that low quality assembly often causes unreliable estimation of *Ka/Ks* [70], we extracted 17 species with relatively better quality (Figure 1) and then removed all in-frame stop codons or gaps in the alignment. According to our age dating information, taxa conflicting with the age were removed. Based on the species tree (Figure 1), we estimated *Ka/Ks* for each branch using free ratio model in PAML [71].

### Functional Enrichment Analysis

We downloaded Gene Ontology (GO) annotations for Ensembl V51. We used the program analyze.pl V1.9 of TermFinder package [72] to identify those significant terms for new genes, with multiple test corrected *p* of 0.05 as the cutoff and the whole genome as the background. Herein, TermFinder was updated to V0.83, which corrected a mistake in calculating false discovery rate [73].

## Supporting Information

Figure S1 **Contribution of each chromosome to genome content.** Each data point shows the proportion of genes originating on a given chromosome out of all genes originating during that evolutionary period, that is, in that phylogenetic branch. Since human and mouse chromosomes are not completely orthologous, we downloaded net chain information (table netMm9) between human and mouse from UCSC [74] and extracted the top mouse hit for each individual human chromosome. For example, the top hit in mouse for human chromosome 1 is mouse chromosome 1. However, it is possible that this pair of chromosomes does not share orthology across their entire lengths.
(0.53 MB DOC)

Figure S2 **The proportions of male-biased genes arising in each evolutionary period for human (Panel A) and mouse (Panel B).** We used the exponential decay formula, *f*(*t*) = *N*(*e^rt^*(*1*−*d*)+*d*), to fit the origination process of male-biased genes, and using maximum likelihood method (nls function in R), estimated the following parameters: *N* = 0.74, *r* = 0.08, and *d* = 0.42 for human and *N* = 0.90, *r* = 0.008, and *d* = 0.22 for mouse. Panel A is based on Affymetrix Research Exon Array data for humans (GSE5791), while panel B is based on the Affymetrix Mouse Exon Array Panel. For the former, since the raw CEL file is not available, we downloaded the processed data from GEO website [28], defined the median value of all exons as the gene-level expression intensity. For the latter, we used aroma.affymetrix package to generate gene-level intensity. Then, we called expression bias using LIMMA package [68]. Here we repeated the analysis in Figure 3 using exon array data because it complements the Affymetrix 3′ gene chip. Notably, Affymetrix 3′ gene chip covers fewer young genes but provides better probe design. In contrast, Affymetrix Exon Array covers many more young genes. However, many genes might be mis-annotated and the average array signal might not reliably reflect gene expression 75. Thus, that two complementary datasets concur provides more convincing evidence for the observed pattern.
(0.11 MB DOC)

Figure S3 **Heatmap of expression for 35 X-linked young genes in different testis cell types.** We generated the figure using gplots package (http://cran.r-project.org/web/packages/gplots/index.html). The top-left figure shows the color key with the histogram of expression intensity embedded.
(0.07 MB DOC)

Figure S4 **Expressional divergence for different cell types in mouse testes.** Since rodent specific genes with unique probes in both mouse and rat are too few, here we define genes emerging since branch 5 as young genes and the remaining entries as old genes.
(0.08 MB DOC)

Figure S5 **The proportions of female-biased genes in all evolutionary periods for human (Panel A) and mouse (Panel B).** The convention follows Figure 3 in the main text. The red arrow marks branch 5 when X chromosome occurred.
(0.12 MB DOC)

Figure S6 **Relative abundance (RA) of nine control tissues in mice.**
(0.55 MB DOC)

Figure S7 **Heatmap of expression enrichment in X chromosome and autosome based on human body index data (GSE7307).** The axes are labeled as in Figure 6 of the main text. Note that branches 10, 11, and 12 were skipped since these branches have too few (<5) genes with unique probes on the X chromosome. Moreover, these data have quite different numbers of replicates for different samples, ranging between 1 and 9 with a median of 4. In this case, we used a stringent criterion for presence, i.e., a gene of interest should be present in all replicates. In all other cases, we simply define them as absent to ensure similar sample size and statistical power.
(0.15 MB DOC)

Figure S8 **Spatial distribution of X-linked genes with respect to branch assignment.** Each gene was marked as one grey point. Local gene density was shown as dashed curves. Evolutionary strata were marked by dashed lines with a yellow circle defining centromere. Based on [46]–[47],[49], pseudoautosomal region (PAR), X-conserved region (XCR), X-added region (XAR), and X-specific region (XSR) were also marked.
(0.12 MB DOC)

Table S1 **Human gene branch assignment together with expression bias annotation.** Branch assignments follow Figure 1A. For genes without unique Affymetrix human 133 plus 2.0 probes, the expression bias is shown as “NA.”
(1.84 MB XLS)

Table S2 **Mouse gene branch assignment together with expression bias annotation.** Branch assignments follow Figure 1B. For genes without unique Affymetrix mouse 430 2.0 probes, the expression bias is shown as “NA.”
(2.01 MB XLS)

Table S3 **Branch assignment comparison between our dataset and** [**10**]. ^a^ The code follows [10]. A, B, C, D, and E refer to the ancestral branch of all mammals, the ancestral branch of eutherian and marsupials, ancestral branch of human, mouse and dog, the ancestral branch of human and mouse, and the branch of the human lineage, respectively. ^b^ Branch specification follows Figure 1A in the main text. ^c^ “Y” indicates the branch assignment is compatible between our work and [10]. “N” indicates we found evidence that this gene should be assigned to older branches. “?” indicates we are not sure which assignment is correct since synteny blocks are very small. ^d^ We checked UCSC synteny information in multiple outgroups and Ensembl orthology information to verify which branch this gene should be assigned to. “Exist in one species” means Ensembl annotates one ortholog in this outgroup. “Micro-synteny” indicates UCSC predicts a small synteny block (covering less than one gene) in this outgroup.
(0.03 MB XLS)

Table S4 **Expression biases of genes originating in branches 6 and 7.** The chi-square test compares whether X chromosome and autosome have different distributions of expression bias.
(0.02 MB XLS)

Table S5 **Estimation of nucleotide substitution driven by positive selection using DoEF package** [**31**] **.** ^a^ Since these re-sequencing data cover so few primate-specific genes (branch≥8), we categorized all eutherian-specific genes (branch≥5) as young genes and the remaining ones as old genes. In other words, all genes originating after the emergence of the X chromosome are defined as “young” genes here. ^b^ LikeLihood Ratio (LLR) test shows whether the estimated α is significantly different compared to the neutral estimation, i.e., α of 0. “ns” indicates “not significant” or *p*≥0.05. ^c^ DoEF did not reach convergence when the sample size is too large. So we randomly sampled 300 genes from all old autosome-linked genes.
(0.02 MB DOC)

Table S6 **miRNA branch assignment and statistics.** (A) Statistical analyses of miRNA gene gain for evolutionary branches where protein-coding genes showed a burst of gene gain. (B) Human miRNA branch assignment. The branch assignments follow Figure 1A. (C) Mouse miRNA branch assignment. The branch convention follows Figure 1B.
(0.15 MB XLS)

Table S7 **The proportion of expressed X-linked miRNAs originating in branch 5 versus proportion of expressed autosomal genes from the same evolutionary period.** Taking placenta as an example, there are 12 X-linked genes present in all replicates and 3 X-linked genes absent in all replicates. In contrast, for autosomal miRNAs, 41 are present and 24 absent in all replicates. So the excess will be 12/15–41/65 or 26.8% (Fisher's Exact Test one-sided *p* = 0.173).
(0.02 MB XLS)

Table S8 **Gene Ontology (GO) enrichment analysis for human X-linked protein-coding genes emerging on branch 5.** “P,” “C,” and “F” indicate three root categories of GO, i.e., biological process, cellular component, and molecular function, respectively.
(0.02 MB XLS)

Table S9 **Expression bias of parental genes and retrogenes.** Since Affymetrix 3′ array does not provide unique probes for many young retrogenes, we used Affymetrix exon array to call sex bias in this table. Cells with female-biased parental gene and male-biased retrogene are marked in red.
(0.02 MB XLS)

Table S10 **Spatial distribution of young X-linked male-biased genes.** “Coordinate” column shows the position for the middle point of each gene. “CT” marks whether a gene is a Cancer/Testis gene or not according to the CTdatabase 76. Notably, “FAM9B” is situated in the undefined region between stratum 3 and 4, and thus we assign “?” for this gene.
(0.02 MB XLS)

Table S11 **Annotation of genes encoded by pseudoautosomal regions (PARs).** “Ensembl Branch” shows the age information inferred from the Ensembl homolog tree [54]. In cases where the tree includes some ambiguous nodes, i.e., Ensembl could not differentiate between speciation events and duplication events, we show “NA” there. Notably, Ensembl annotated 30 entries in PARs, 10 of which are pseudogenes. For the remaining 20 genes, we dated 17 entries.
(0.02 MB XLS)

## Abbreviations

CNP: copy number polymorphism
CT: cancer/testis
myr: million years
PAR: pseudoautosomal region
RA: relative abundance
XAR: X-added region
XCR: X-conserved region
