# Supplementary material for: Chromosomal Redistribution of Male-Biased Genes in Mammalian Evolution with Two Bursts of Gene Gain on the X Chromosome
Source: PLoS Biol. 2010 Oct 5;8(10):e1000494. doi: 10.1371/journal.pbio.1000494 (PMC2950125; doi:10.1371/journal.pbio.1000494)

**Figure S1.** Contribution of each chromosome to genome content. Each data point shows the proportion of genes originating on a given chromosome out of all genes originating during that evolutionary period, that is, in that phylogenetic branch. Since human and mouse chromosomes are not completely orthologous, we downloaded net chain information (table netMm9) between human and mouse from UCSC [5] and extracted the top mouse hit for each individual human chromosome. For example, the top hit in mouse for human chromosome 1 is mouse chromosome 1. However, it is possible that this pair of chromosomes does not share orthology across their entire lengths.


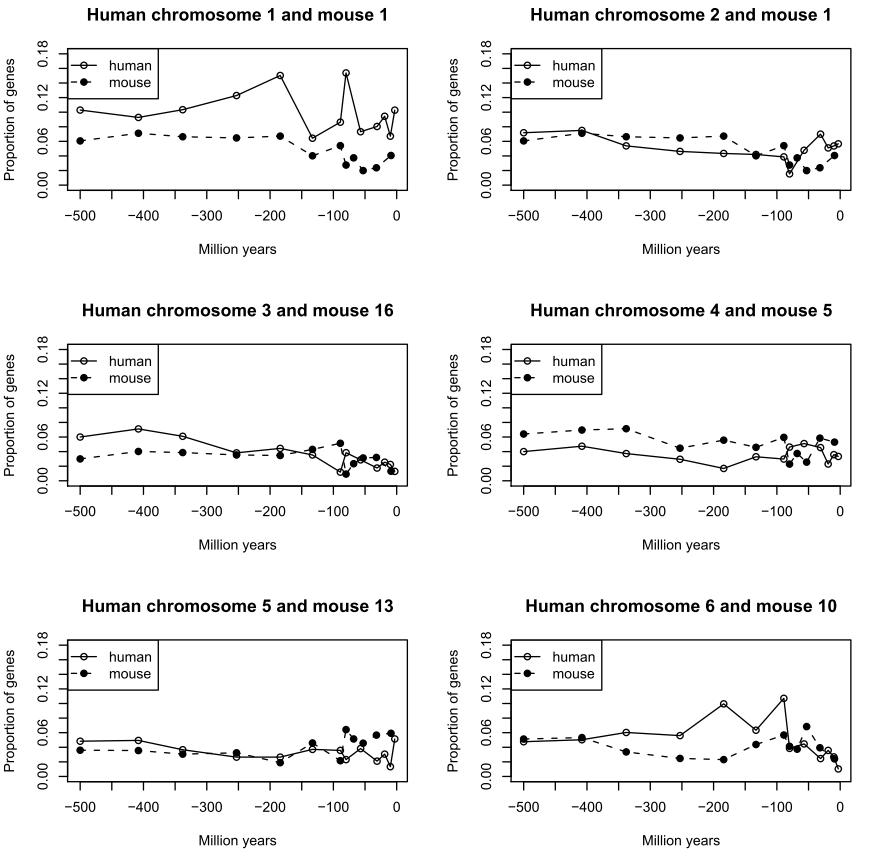


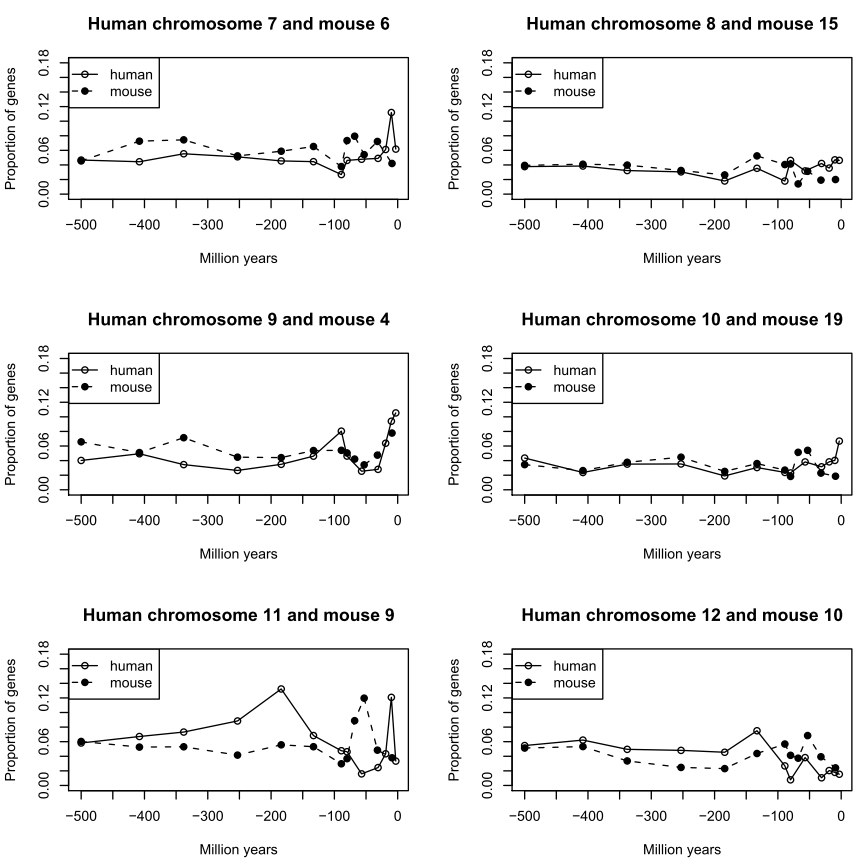


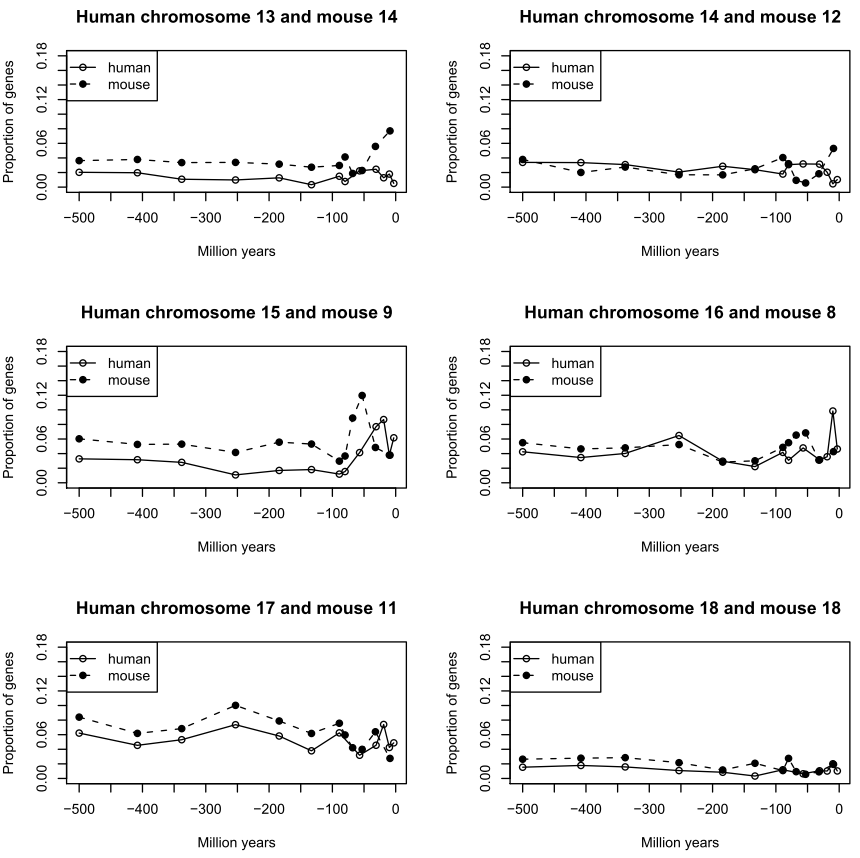


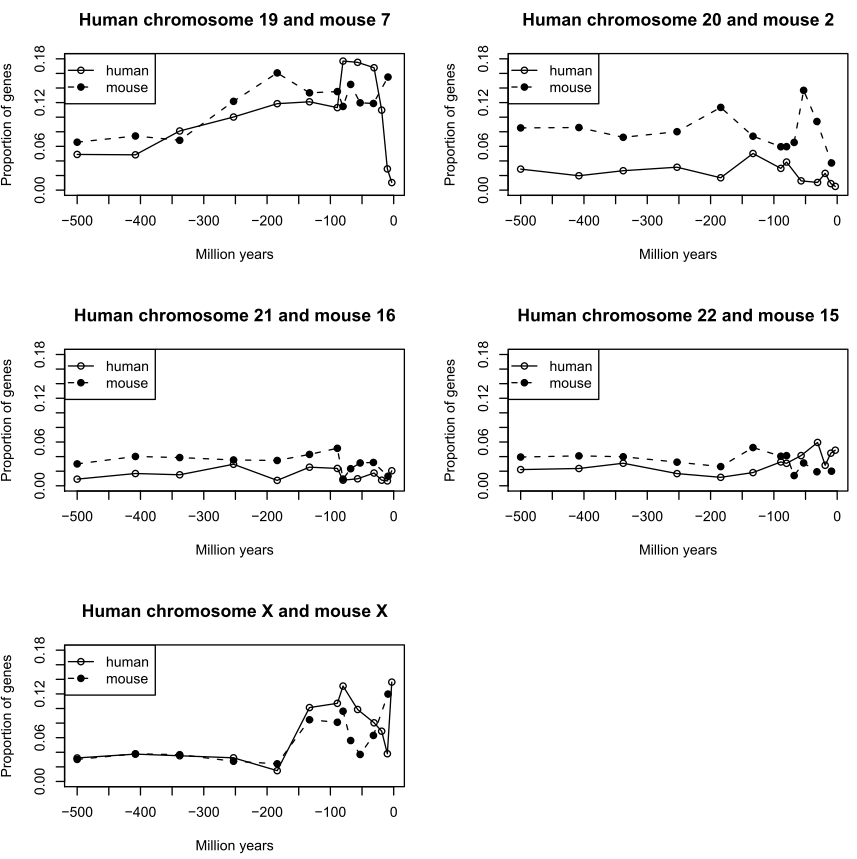

Supplement: Figure S1 — Contribution of each chromosome to genome content. Each data point shows the proportion of genes originating on a given chromosome out of all genes originating during that evolutionary period, that is, in that phylogenetic branch. Since human and mouse chromosomes are not completely orthologous, we downloaded net chain information (table netMm9) between human and mouse from UCSC [74] and extracted the top mouse hit for each individual human chromosome. For example, the top hit in mouse for human chromosome 1 is mouse chromosome 1. However, it is possible that this pair of chromosomes does not share orthology across their entire lengths. (0.53 MB DOC) [file pbio.1000494.s001.doc]
