# Supplementary material for: Chromosomal Redistribution of Male-Biased Genes in Mammalian Evolution with Two Bursts of Gene Gain on the X Chromosome
Source: PLoS Biol. 2010 Oct 5;8(10):e1000494. doi: 10.1371/journal.pbio.1000494 (PMC2950125; doi:10.1371/journal.pbio.1000494)
Supplement: Figure S2 — The proportions of male-biased genes arising in each evolutionary period for human (Panel A) and mouse (Panel B). We used the exponential decay formula, f(t) = N(ert(1−d)+d), to fit the origination process of male-biased genes, and using maximum likelihood method (nls function in R), estimated the following parameters: N = 0.74, r = 0.08, and d = 0.42 for human and N = 0.90, r = 0.008, and d = 0.22 for mouse. Panel A is based on Affymetrix Research Exon Array data for humans (GSE5791), while panel B is based on the Affymetrix Mouse Exon Array Panel. For the former, since the raw CEL file is not available, we downloaded the processed data from GEO website [28], defined the median value of all exons as the gene-level expression intensity. For the latter, we used aroma.affymetrix package to generate gene-level intensity. Then, we called expression bias using LIMMA package [68]. Here we repeated the analysis in Figure 3 using exon array data because it complements the Affymetrix 3′ gene chip. Notably, Affymetrix 3′ gene chip covers fewer young genes but provides better probe design. In contrast, Affymetrix Exon Array covers many more young genes. However, many genes might be mis-annotated and the average array signal might not reliably reflect gene expression 75. Thus, that two complementary datasets concur provides more convincing evidence for the observed pattern. (0.11 MB DOC) [file pbio.1000494.s002.doc]

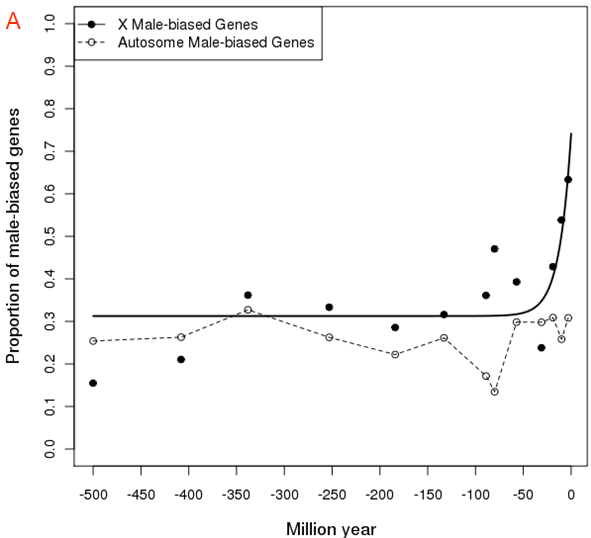

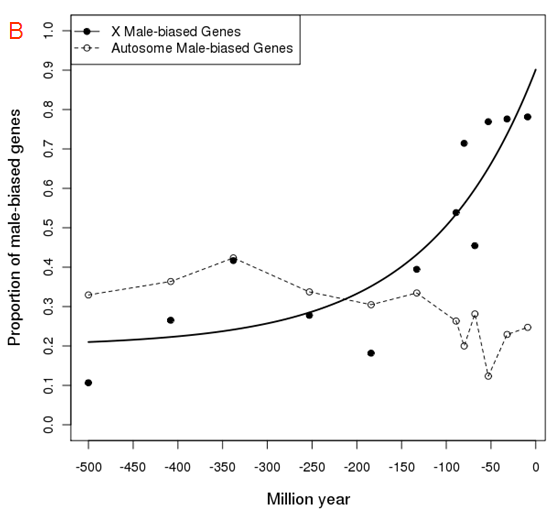


**Figure S2.** The proportions of male-biased genes arising in each evolutionary period for human (Panel A) and mouse (Panel B). We used the exponential decay formula, *f*(*t*)*=N*(*e^rt^*(*1-d*)*+d*), to fit the origination process of male-biased genes, and, using maximum likelihood method (nls function in R), estimated the following parameters: *N*=0.74, *r*=0.08 and *d*=0.42 for human and *N*=0.90, *r*=0.008 and *d*=0.22 for mouse.

Panel A is based on Affymetrix Research Exon Array data for humans (GSE5791), while panel B is based on the Affymetrix Mouse Exon Array Panel. For the former, since the raw CEL file is not available, we downloaded the processed data from GEO website [6], defined the median value of all exons as the gene-level expression intensity. For the later, we used aroma.affymetrix package to generate gene-level intensity. Then, we called expression bias using LIMMA package [7].

Here we repeated the analysis in Figure 3 using exon array data because it complements the Affymetrix 3’ gene chip. Notably, Affymetrix 3’ gene chip covers fewer young genes, but provides better probe design. In contrast, Affymetrix Exon Array covers many more young genes. However, many genes might be mis-annotated and the average array signal might not reliably reflect gene expression [8]. Thus, that two complementary datasets concur provides more convincing evidence for the observed pattern.
