# Supplementary material for: Chromosomal Redistribution of Male-Biased Genes in Mammalian Evolution with Two Bursts of Gene Gain on the X Chromosome
Source: PLoS Biol. 2010 Oct 5;8(10):e1000494. doi: 10.1371/journal.pbio.1000494 (PMC2950125; doi:10.1371/journal.pbio.1000494)
Supplement: Figure S3 — Heatmap of expression for 35 X-linked young genes in different testis cell types. We generated the figure using gplots package (http://cran.r-project.org/web/packages/gplots/index.html). The top-left figure shows the color key with the histogram of expression intensity embedded. (0.07 MB DOC) [file pbio.1000494.s003.doc]

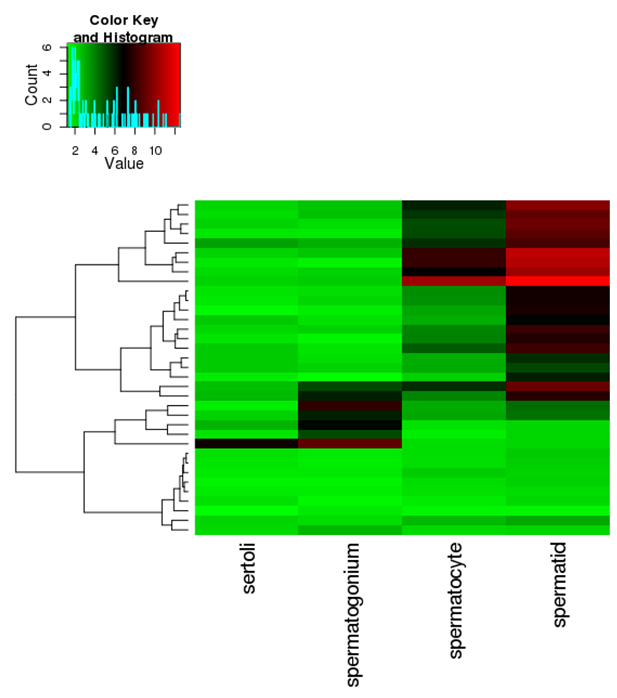


**Figure S3.** Heatmap of expression for 35 X-linked young genes in different testis cell types. We generated the figure using gplots package (<http://cran.r-project.org/web/packages/gplots/index.html>). The top-left figure shows the color key with the histogram of expression intensity embedded.
