# Supplementary material for: Chromosomal Redistribution of Male-Biased Genes in Mammalian Evolution with Two Bursts of Gene Gain on the X Chromosome
Source: PLoS Biol. 2010 Oct 5;8(10):e1000494. doi: 10.1371/journal.pbio.1000494 (PMC2950125; doi:10.1371/journal.pbio.1000494)
Supplement: Figure S4 — Expressional divergence for different cell types in mouse testes. Since rodent specific genes with unique probes in both mouse and rat are too few, here we define genes emerging since branch 5 as young genes and the remaining entries as old genes. (0.08 MB DOC) [file pbio.1000494.s004.doc]

| 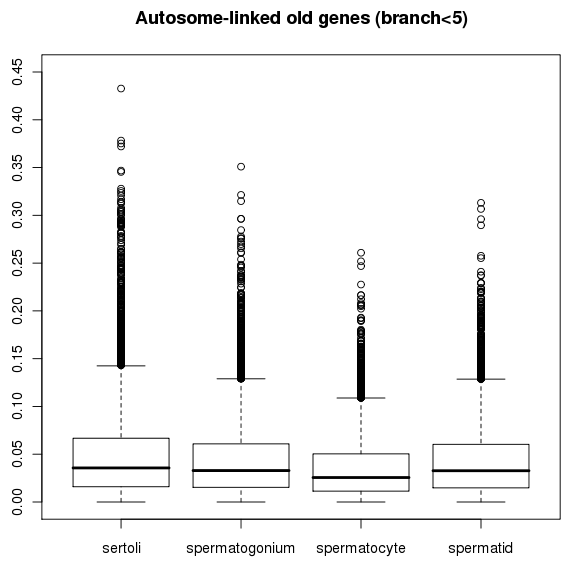 | 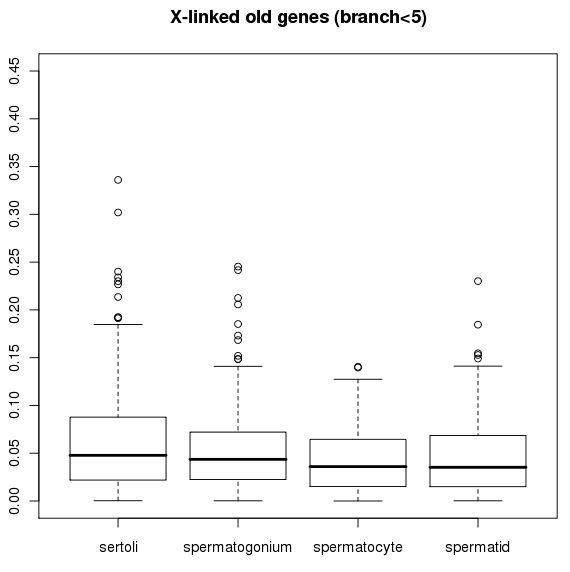 |
| --- | --- |

**Figure S4.** Expressional divergence for different cell types in mouse testis. Since rodent specific genes with unique probes in both mouse and rat are too few, here we define genes emerging since branch 5 as young genes and the remaining entries as old genes.
