# Supplementary material for: Chromosomal Redistribution of Male-Biased Genes in Mammalian Evolution with Two Bursts of Gene Gain on the X Chromosome
Source: PLoS Biol. 2010 Oct 5;8(10):e1000494. doi: 10.1371/journal.pbio.1000494 (PMC2950125; doi:10.1371/journal.pbio.1000494)
Supplement: Figure S5 — The proportions of female-biased genes in all evolutionary periods for human (Panel A) and mouse (Panel B). The convention follows Figure 3 in the main text. The red arrow marks branch 5 when X chromosome occurred. (0.12 MB DOC) [file pbio.1000494.s005.doc]

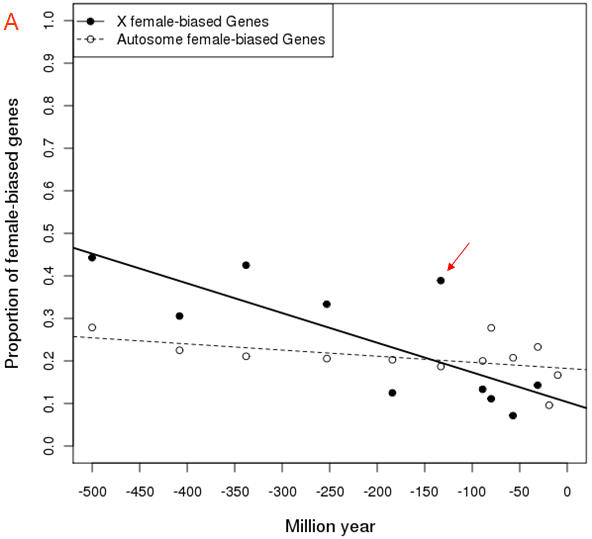

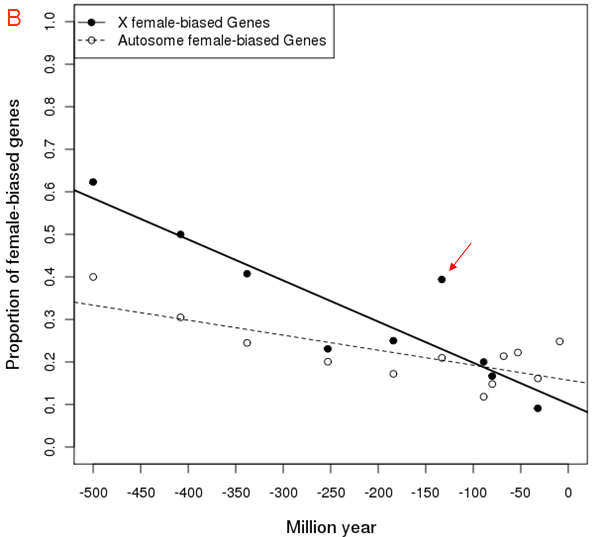


**Figure S5.** The proportions of female-biased genes in all evolutionary periods for human (Panel A) and mouse (Panel B). The convention follows Figure 3 in the main text. The red arrow marks branch 5 when X chromosome occurred.
