# Supplementary figures and images for: Chromosomal Redistribution of Male-Biased Genes in Mammalian Evolution with Two Bursts of Gene Gain on the X Chromosome
Source: PLoS Biol. 2010 Oct 5;8(10):e1000494. doi: 10.1371/journal.pbio.1000494 (PMC2950125; doi:10.1371/journal.pbio.1000494)

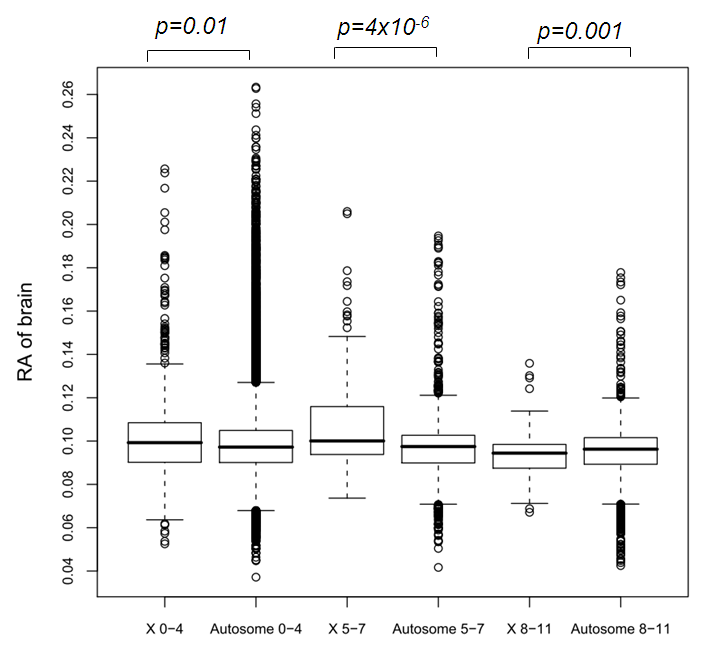

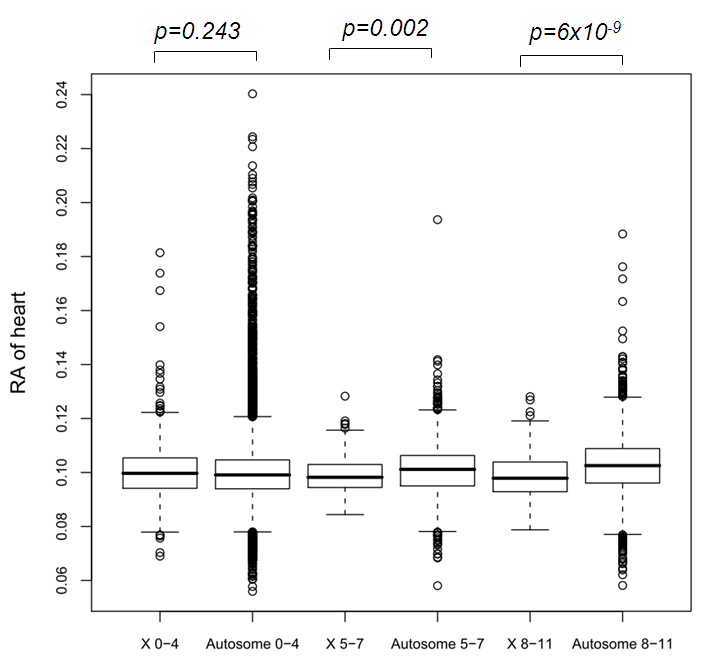


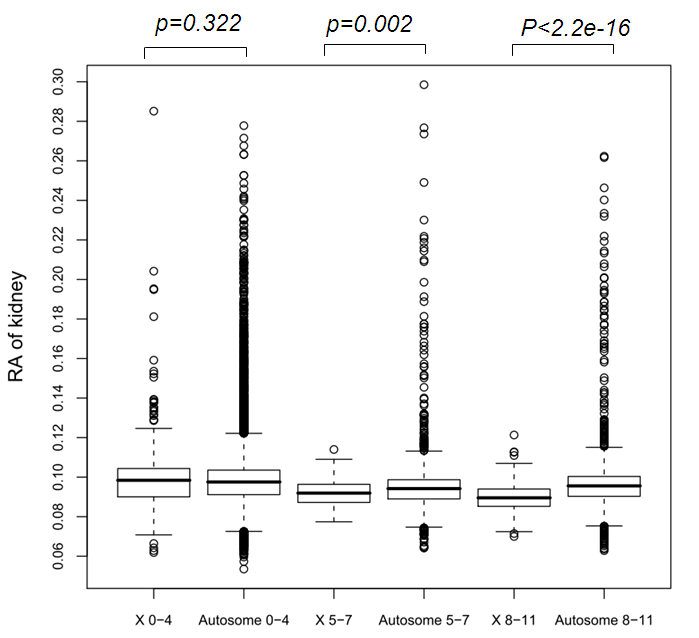

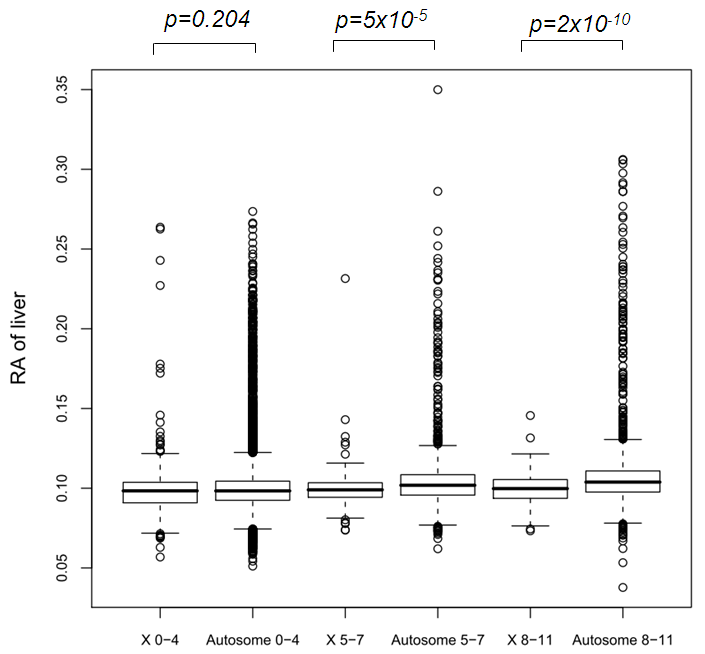


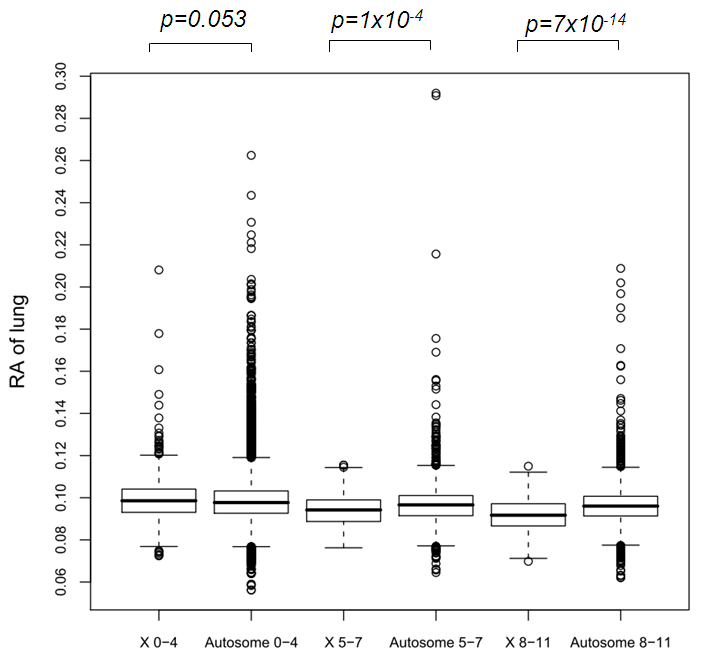

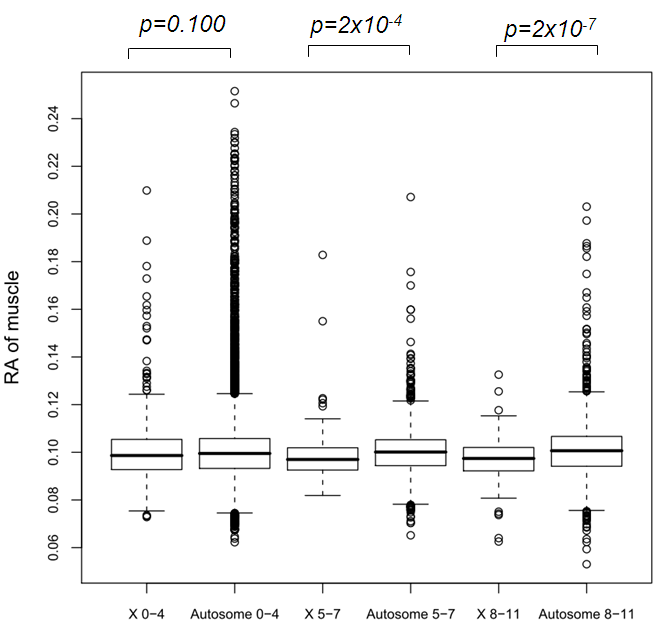


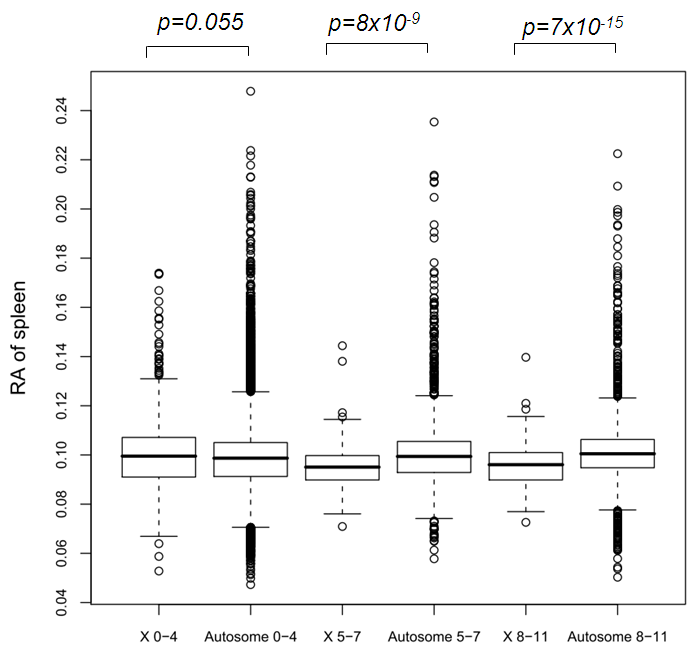

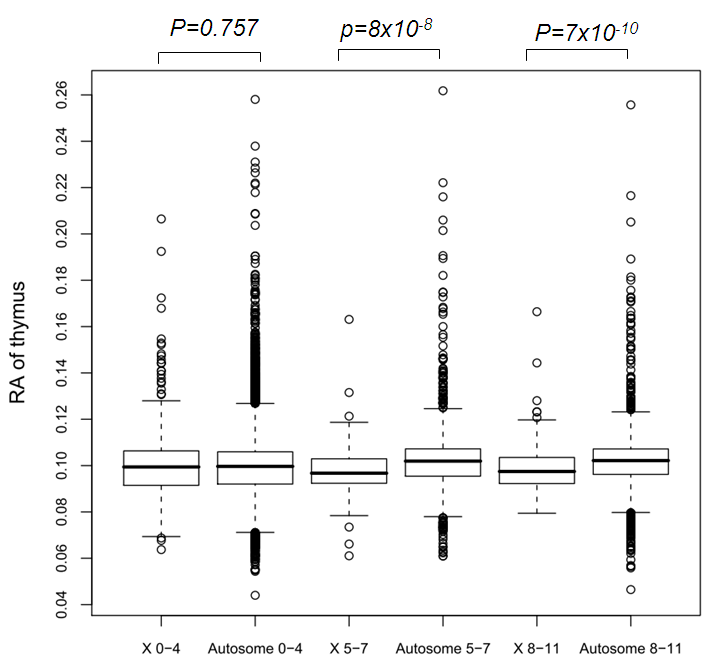


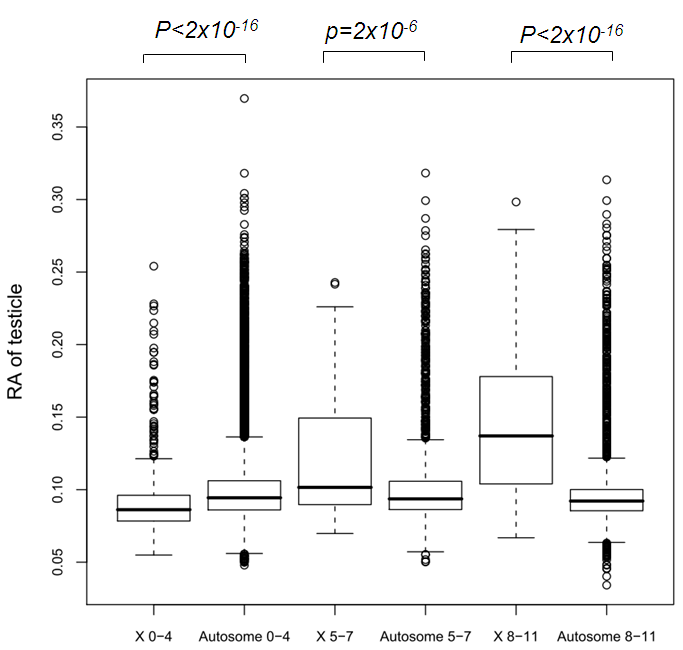


**Figure S6.** Relative abundance (RA) of nine control tissues in mice

Supplement: Figure S6 — Relative abundance (RA) of nine control tissues in mice. (0.55 MB DOC) [file pbio.1000494.s006.doc]
