# Supplementary material for: Chromosomal Redistribution of Male-Biased Genes in Mammalian Evolution with Two Bursts of Gene Gain on the X Chromosome
Source: PLoS Biol. 2010 Oct 5;8(10):e1000494. doi: 10.1371/journal.pbio.1000494 (PMC2950125; doi:10.1371/journal.pbio.1000494)
Supplement: Figure S7 — Heatmap of expression enrichment in X chromosome and autosome based on human body index data (GSE7307). The axes are labeled as in Figure 6 of the main text. Note that branches 10, 11, and 12 were skipped since these branches have too few (<5) genes with unique probes on the X chromosome. Moreover, these data have quite different numbers of replicates for different samples, ranging between 1 and 9 with a median of 4. In this case, we used a stringent criterion for presence, i.e., a gene of interest should be present in all replicates. In all other cases, we simply define them as absent to ensure similar sample size and statistical power. (0.15 MB DOC) [file pbio.1000494.s007.doc]

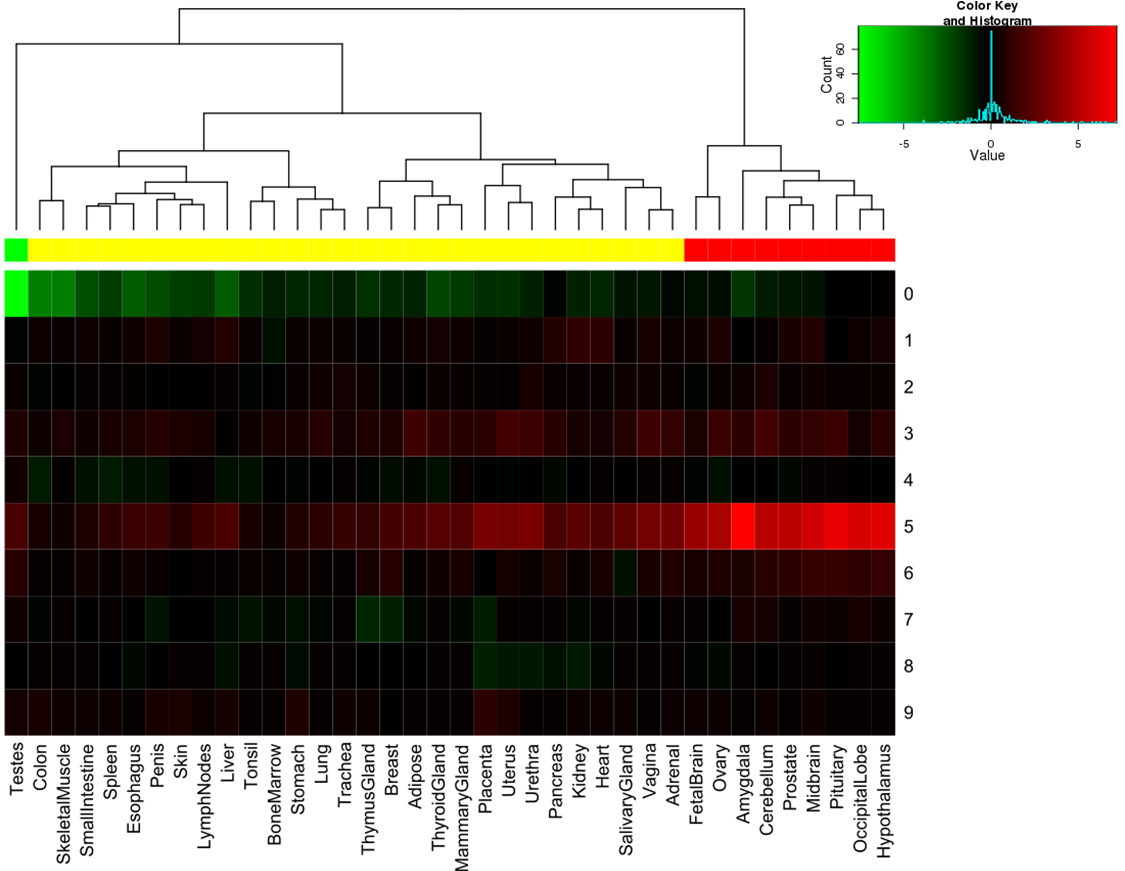

**Figure S7.** Heatmap of expression enrichment in X chromosome and autosome based on human body index data (GSE7307). The axes are labeled as in Figure 6 of the main text. Note that branches 10, 11 and 12 were skipped since these branches have too few (<5) genes with unique probes on the X chromosome. Moreover, this data has quite different numbers of replicates for different samples, ranging between 1 and 9 with a median of 4. In this case, we used a stringent criterion for presence, *i.e.* a gene of interest should be present in all replicates. In all other cases, we simply define them as absent to ensure similar sample size and statistical power.
