# Supplementary material for: Chromosomal Redistribution of Male-Biased Genes in Mammalian Evolution with Two Bursts of Gene Gain on the X Chromosome
Source: PLoS Biol. 2010 Oct 5;8(10):e1000494. doi: 10.1371/journal.pbio.1000494 (PMC2950125; doi:10.1371/journal.pbio.1000494)
Supplement: Figure S8 — Spatial distribution of X-linked genes with respect to branch assignment. Each gene was marked as one grey point. Local gene density was shown as dashed curves. Evolutionary strata were marked by dashed lines with a yellow circle defining centromere. Based on [46]–[47],[49], pseudoautosomal region (PAR), X-conserved region (XCR), X-added region (XAR), and X-specific region (XSR) were also marked. (0.12 MB DOC) [file pbio.1000494.s008.doc]

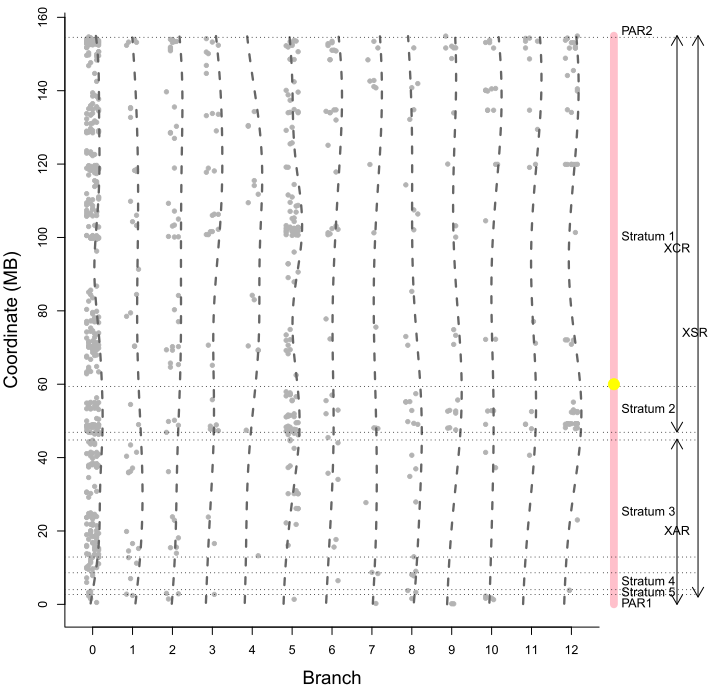


**Figure S8.** Spatial distribution of X-linked genes with respect to branch assignment. Each gene was marked as one grey point. Local gene density was shown as dashed curves. Evolutionary strata were marked by dashed lines with yellow circle defining centromere. Based on [9-11], pseudoautosomal region (PAR), X-conserved region (XCR), X-added region (XAR) and X-specific region (XSR) were also marked.
